# Therapeutic targeting of chronic kidney disease-associated DAMPs differentially contributing to vascular pathology

**DOI:** 10.3389/fimmu.2023.1240679

**Published:** 2023-10-02

**Authors:** Morgane Mazzarino, Esra Cetin, Maria Bartosova, Iva Marinovic, Natacha Ipseiz, Timothy R. Hughes, Claus Peter Schmitt, Dipak P. Ramji, Mario O. Labéta, Anne-Catherine Raby

**Affiliations:** ^1^ Division of Infection & Immunity, Cardiff University, Cardiff, United Kingdom; ^2^ Wales Kidney Research Unit, School of Medicine, Cardiff University, Cardiff, United Kingdom; ^3^ Division of Pediatric Nephrology, Center for Pediatric and Adolescent Medicine, University Hospital Heidelberg, Heidelberg, Germany; ^4^ School of Biosciences, Cardiff University, Cardiff, United Kingdom

**Keywords:** chronic kidney disease, vascular inflammation, damage-associated molecular patterns (DAMPs), toll-like receptors (TLRs), anti-inflammatory intervention strategies

## Abstract

Chronic Kidney Disease (CKD) is associated with markedly increased cardiovascular (CV) morbidity and mortality. Chronic inflammation, a hallmark of both CKD and CV diseases (CVD), is believed to drive this association. Pro-inflammatory endogenous TLR agonists, Damage-Associated Molecular Patterns (DAMPs), have been found elevated in CKD patients’ plasma and suggested to promote CVD, however, confirmation of their involvement, the underlying mechanism(s), the extent to which individual DAMPs contribute to vascular pathology in CKD and the evaluation of potential therapeutic strategies, have remained largely undescribed. A multi-TLR inhibitor, soluble TLR2, abrogated chronic vascular inflammatory responses and the increased aortic atherosclerosis-associated gene expression observed in nephropathic mice, without compromising infection clearance. Mechanistically, we confirmed elevation of 4 TLR DAMPs in CKD patients’ plasma, namely Hsp70, Hyaluronic acid, HMGB-1 and Calprotectin, which displayed different abilities to promote key cellular responses associated with vascular inflammation and progression of atherosclerosis in a TLR-dependent manner. These included loss of trans-endothelial resistance, enhanced monocyte migration, increased cytokine production, and foam cell formation by macrophages, the latter via cholesterol efflux inhibition. Calprotectin and Hsp70 most consistently affected these functions. Calprotectin was further elevated in CVD-diagnosed CKD patients and strongly correlated with the predictor of CV events CRP. In nephropathic mice, Calprotectin blockade robustly reduced vascular chronic inflammatory responses and pro-atherosclerotic gene expression in the blood and aorta. Taken together, these findings demonstrated the critical extent to which the DAMP-TLR pathway contributes to vascular inflammatory and atherogenic responses in CKD, revealed the mechanistic contribution of specific DAMPs and described two alternatives therapeutic approaches to reduce chronic vascular inflammation and lower CV pathology in CKD.

## Introduction

Chronic Kidney Disease (CKD) is a growing public health problem, affecting up to 15% of the population (1, 2). CKD at all stages is associated with significantly increased cardiovascular (CV) morbidity and mortality (3, 4), with patients on dialysis showing a 20-fold increase in CV mortality (3).

Traditional risk factors do not account for the higher CV risk in CKD, and clinical interventions successful in the general CV population are not always effective in CKD patients. CKD-specific factors appear to be partially responsible (3, 4). The chronic inflammation associated with kidney damage and the dialysis treatment has been suggested to play a substantial role in promoting CV diseases (CVD), such as atherosclerosis, which may lead to peripheral vascular disease (PVD) or coronary artery disease (CAD) (3, 4). In line with this, higher plasma levels of the inflammatory markers C-reactive protein (CRP) and IL-6 can predict cardiovascular events and mortality in CKD patients (5–8). Notably, antibody blocking of the pro-inflammatory cytokine IL-1β reduced the risk of major adverse cardiovascular events among those with CKD (9, 10), demonstrating the potential of reducing chronic inflammation to reduce CV risk. However, overall mortality was not reduced by this strategy, as IL-1β inhibition significantly increased the risk of fatal infections (9). Therefore, a better understanding of the specific pathway(s) involved in driving chronic inflammation in CKD is required to develop alternative safer anti-inflammatory treatment options.

Critical to the development of chronic inflammatory pathologies, including CV pathologies such as atherosclerosis, are the Toll-like receptors (TLRs) (11, 12). They play a central role in mediating inflammation in response to infection or tissue damage, the latter via recognition of endogenous ligands, Damage-Associated Molecular Patterns (DAMPs), released following tissue injury (13). TLR DAMPs have been detected in endarterectomy samples, notably High-Mobility Group Box 1 (HMGB1, a TLR2/TLR4 agonist) and Calprotectin (S100A8/S100A9, a TLR4 agonist), and are associated with plaque progression and destabilisation (14–16). Plasma levels of Heat-shock protein (Hsp) 60 and 70 (TLR2 and TLR4 ligands) and Calprotectin can predict CV events in the general population (16–19). Notably, CKD-associated tissue injury results in TLR DAMPs production in the kidney (20), and elevated urinary DAMP levels may serve as biomarkers of kidney damage (21–23). Furthermore, elevated levels of circulating DAMPs including, Calprotectin (24), S100A12 (25) and mitochondrial DNA (26) were also reported in patients on hemodialysis.

Although DAMPs’ involvement in CV risk in CKD has been suggested (27), confirmation of their involvement, the underlying mechanism(s), the extent to which individual DAMPs contribute to vascular pathology in CKD, and the evaluation of potential therapeutic strategies, have remained largely undescribed. In this study, we demonstrated that the DAMP-TLR pathway is a major contributor to systemic inflammatory and vascular responses that drive CVD during chronic nephropathy, and that it can be efficiently targeted using a multi-TLR inhibitor, an approach that does not compromise bacterial clearance. We confirmed the elevation of 4 TLR DAMPs in late-stage CKD patients namely, Hsp70, Hyaluronic acid (HA), HMGB-1 and Calprotectin, and extended this finding by assessing the contribution of each DAMP to vascular pathological responses. We found that they differentially promoted key cellular functions and responses in endothelial cells, monocytes and macrophages associated with vascular inflammation and dysfunction and atherosclerosis promotion. Of the 4 TLR DAMPs found elevated in late-stage CKD, Calprotectin and Hsp70 most robustly and consistently affected the functions tested. Calprotectin was further elevated in CVD-diagnosed CKD patients, highly correlated with the predictor of CV events CRP, and its pharmacologic inhibition substantially reduced the vascular consequences of chronic nephropathy in mice. Thus, this study provides mechanistic confirmation of the contribution of specific TLR DAMPs in driving vascular inflammation and atherosclerosis-promoting responses in CKD and demonstrates the therapeutic potential of multi-TLR- and specific DAMP-targeting strategies to lower CV risk in CKD.

## Methods

### Human blood samples

Blood samples from Stage 5 CKD patients at PD initiation time and healthy individuals were obtained in accordance with the institutional review board of Cardiff University and the local National Health Service Research Ethics Committee (**Table 1**). Written informed consent was obtained from all donors. Plasma samples were separated by centrifugation (15 min, 1300 x g), aliquoted (200 µl) and promptly frozen at -80°C until testing. Samples from CKD patients with diabetes or malignancy were excluded from the analysis. CVD diagnosis of CKD patients included ischemic heart disease and peripheral vascular disease (Table 2).

**Table 1:** Healthy donors, Stage 5 CKD and Stage 5 CKD + CVD patients characteristics at sampling time.

	Healthy	CKD Stage 5	CKD Stage 5 + CVD	*p* value (Healthy vs CKD Stage 5)	*p* value (CKD Stage 5 vs CKD Stage 5 + CVD)
Number	30	37	37	–	–
Age, yr: Median (interquartile range)	48 (28–54)	50 (30-58)	67 (62-71)	0.354*	<0.0001*
Female sex: n (%)	11 (37)	12 (32)	9 (24)	0.716^#^	0.439^#^
Ischemic heart disease (IHD): n (%)	0 (0)	0 (0)	16 (43)	–	–
Peripheral vascular disease (PVD): n (%)	0 (0)	0 (0)	8 (22)	–	–
IHD + PVD: n (%)	0 (0)	0 (0)	13 (35)	–	–

Mann-Whitney U test* and chi-square test^#^ were used to test for differences between groups.

### Cell culture

Human aortic endothelial cells (HAEC, NBS Biologicals) were cultured in M200 supplemented with Large Vessel Endothelial Supplement (Fisher Scientific). Mono-Mac6 (MM6) cells were cultured in RPMI 1640 medium supplemented with 10% low endotoxin fetal calf serum (FCS, HyClone; < 0.06 U/mL endotoxin), 1% insulin (Fisher Scientific), 1% Non-essential Amino Acids (Fisher Scientific) and 1% pyruvate (Fisher Scientific).

Human peripheral blood mononuclear cells (PBMC) were obtained from buffy coats (Welsh Blood Services) through Ficoll density-gradient centrifugation and cultured in RPMI 1640 medium supplemented with 10% low endotoxin FCS. Macrophages were differentiated from blood monocytes obtained by PBMC adhesion (2h, RMPI 1640 supplemented with 1% FCS). Non-adherent cells were removed, and adherent monocytes were rinsed (3 times, PBS) before culture for at least 7 days in complete medium supplemented with human M-CSF (10 ng/ml, Peprotech). The culture medium was then supplemented with fresh M-CSF every 3 days for the remainder of the culture.

### Functional assays

For activation experiments, triplicate aliquots of HAEC (5 x 10^3^ cells/well), PBMC (1 x 10^5^ cells/well) or M-CSF differentiated macrophages (1.5 x 10^4^ cells/well) were cultured (18h) in the presence of the indicated concentrations of ultra-pure LPS (E. coli O111:B4 strain; Invivogen), the synthetic bacterial lipopeptide Pam_3_-CSK_4_ (EMC microcollections), recombinant human Hsp70 (active, functional grade, Abcam), recombinant human Calprotectin (S100A8/A9, functional grade, Biolegend), recombinant human HMGB-1 (functional grade, R&D), hyalorunic acid (HA, low and medium molecular weight, functional grade, R&D), or a combination of the 4 DAMPs. For TLR blocking experiments, cells were pre-incubated (30 min) with neutralising mAbs (10 µg/ml), alone or in combination: anti-TLR2 (clone T2.5, Invivogen) and anti-TLR4 (clone 3C3, Invivogen), or with an isotype control (clone MOPC21) prior to addition of the indicated concentrations of TLR ligands. Cell viability was routinely assessed at the end of the experiments by Trypan Blue staining and was always > 90%.

### Focused transcriptomic analyses

Total RNA was extracted from mouse blood (QIAamp RNA Blood Mini Kit) or mouse aortas (Qiagen, RNeasy Fibrous Tissue Mini Kit) following the manufacturer’s instructions, quantified by Nanodrop, and kept frozen (-80°C) until further use. Purified RNA (250 ng/condition) was converted into cDNA by reverse transcription (RT^2^ First Strand kit, Qiagen). Focused transcriptomic analysis was then performed by quantitative PCR of the cDNA using an Atherosclerosis or Innate and Adaptive responses RT^2^ Profiler PCR Array (Qiagen), following the manufacturer’s instructions. Analysis was perfomed automatically via the Qiagen Geneglobe analysis tool. Reference genes were selected automatically from the housekeeping gene panel and relative gene expression was calculated using the ΔΔCt method.

### Flow cytometry

Expression levels of cell surface antigens were determined by flow cytometry as previously described (28). Briefly, cell suspensions (0.15-1 x 10^6^ cells/staining or 30 µl of red-blood cell lysed mouse blood) were prepared in sterile PBS and Fc receptors were blocked by incubation with 50% normal rabbit serum (10 min, RT) or mouse Fc block (for mouse blood, BD Biosciences) before incubation (45 min, 4°C) with directly conjugated monoclonal antibodies (all from BioLegend). Cells were rinsed twice with PBS before immediate flow cytometry analysis on an Attune NxT cytometer. Cellular debris and doublets were excluded based on their FSC-A/SSC-A and FSC-H/FSC-A scatter profiles, respectively.

HAEC cells were stained for ICAM-1 (clone HA58-FITC) and VCAM-1 (clone STA-APC), MM6 cells for CCR2 (clone K036C2-BV421) and macrophage cultures for SR-A (clone 7C9C20-APC) and CD36 (clone 5-271- FITC), following the stimulatory conditions indicated in the text.

For *in vivo* experiments, mouse blood samples underwent red blood cell lysis (Pharm Lyse, BD Biosciences) prior to staining for Ly6C (clone HK1.4-AF488), Ly6G (clone 1A8-AF647), CD11b (clone M1/70-PerCP Cy5.5) and PSLG-1 (clone 2PH1-BV421). Neutrophils were defined as Ly6G^+^ and CD11b^+^ and monocytes as Ly6G^-^ and CD11b^+^. Among total monocytes, Ly6C^high^ and Ly6C ^low^ populations were determined based on their Ly6C expression.

### Trans-endothelial resistance measurements

Trans-endothelial electrical resistance (TER) was measured as previously described (29). Briefly, primary human umbilical arterial endothelial cells (HUAEC, PromoCell) were seeded on 0.4 µm polyester transwell-inserts placed in 24 well plates (50,000 cells/insert, low serum cell growth medium). TER was measured daily with an EVOM volt/ohm meter with STX-2 electrodes (World Precision Instruments) until it reached a plateau, indicating the formation of a confluent, well-polarised monolayer. Baseline TER was measured, and medium was exchanged for treatment solution containing the indicated concentrations of DAMPs, alone or in combination. TER was then measured at the indicated time points. Plotted TER values were calculated by subtracting blank well measurement from control wells with cells and multiplying it by 0.33 (area) with resulting unit of Ω.cm^2^. A ratio was calculated between the TER values after the treatment at each time points and the baseline TER (0h) values to calculate a % of initial TER for each condition.

For ZO-1 visualisation, cells were fixed with 100% EtOH for (5 min, -20°C), followed by permeabilisation with 0.5% TritonX (10 min, RT). Cells were then incubated with 5% BSA for 60 min at room temperature before staining with an AF555-conjugated anti-ZO-1 mAb (Invitrogen, MA3-39100-A555, 1:500, 1h, RT). Cells were washed three times and nuclei were stained with DAPI (Invitrogen, 30 nM, 15min, RT). Transwell filters were cut out from the plastic and fixed in Prolong Gold (Thermo Fisher, 10ul) on a glass slide. Slides were allowed to harden for 24h. Cells were visualized using Acquifer widefield microscope (ACQUIFER Imaging GmbH). For each condition, z-stack images (10 slices, 3 μm slice distance) were acquired using a 20X objective. The Fiji software was used for image analysis. Greyscale images were used to create z-stack projections using maximum intensity method to obtain a clear signal from cell membrane areas. Three stacks were used for every condition. Five different areas were randomly selected for each condition where the cell membranes were annotated manually, and ZO-1 staining intensity was measured at annotated cell-to-cell contacts and corrected for analysed area to obtain the values shown.

### Monocyte migration

MM6 cells (1 x10^6^ cells/well) were cultured (18h, 37°C) in the presence or absence of ultra-pure LPS (10 ng/ml), recombinant human Hsp70 (1 µg/ml), recombinant human Calprotectin (1 µg/ml), recombinant human HMGB-1 (1 µg/ml), HA (low and medium molecular weight, 500 ng/ml each) or a combination of all DAMPs. Cells were then starved (1h) in serum-free medium prior to seeding (200,000 cells, in triplicates) in the top chamber of 8 µm pores transwells. The bottom compartment was filled with RPMI containing 10% FCS and recombinant human MCP-1 (CCL2, functional grade, R&D, 50ng/ml). Cell numbers were counted in the bottom compartment at the indicated time points (typically 2h, 4h, 6h and/or 24h).

### Foam cell formation

Following a starvation step (24h, no serum, RPMI 1640 supplemented with 0.2% fatty-acid free BSA), macrophages were seeded in 8-well microscopy slide (175,000 cells/well) and cultured (24h, no serum) in the presence, or not, of LDL (Invitrogen, 25 µg/ml) alone or together with recombinant human Hsp70 (1 µg/ml), recombinant human Calprotectin (1 µg/ml), recombinant human HMGB-1 (1 µg/ml), HA (low and medium molecular weight, 500 ng/ml each) or a combination of all DAMPs. Intracellular neutral lipids were visualised by Oil Red-O staining (30 min, 37°C), as previously described (30). Slides were mounted using DAPI mounting medium (Fluoroshield, Abcam) to preserve fluorescence and visualise cell nucleus. Images (20x magnification) of 5 non-overlapping fields of view were taken for each condition on a Leica DM LA microscope. To quantify foam cell formation, 20 cells were selected from the top left corner of each image, each cell with detectable red staining was considered a foam cell, while cells without staining were considered normal cells. The process was repeated with the 5 images taken for each condition (total of 100 cells counted/condition). The percentage of foam cells per condition is shown as a measure of foam cell formation.

### Cholesterol uptake and efflux

For modified LDL uptake experiments, macrophages were seeded in 24-well plates (40,000 cells/well), and starved in serum-free medium overnight (RPMI 1640 supplemented with 0.2% fatty-acid free BSA), in the presence or absence of ultra-pure LPS (10 ng/ml), recombinant human Hsp70 (1 µg/ml), recombinant human Calprotectin (1 µg/ml), recombinant human HMGB-1 (1 µg/ml), HA (low and medium molecular weight, 500 ng/ml each) or a combination of all DAMPs. Dil (554/571)-conjugated acetylated LDL (Dil-AcLDL, Alfa Aesar) was added to the culture (10 µg/ml, 18h) and internalised Dil-AcLDL was quantified by flow cytometry (20,000 events/condition, MFI shown).

For efflux experiments, macrophages were seeded in 24-well plates (40,000 cells/well) and loaded with BODIPY-labelled cholesterol (RMPI 1640 supplemented with 0.2% fatty-acid free BSA, 5 µM, 18h), before medium removal and exposure to recombinant human Hsp70 (1 µg/ml), recombinant human Calprotectin (1 µg/ml), recombinant human HMGB-1 (1 µg/ml), HA (low and medium molecular weight, 250 ng/ml each) or a combination of all DAMPs. After equilibration (1h), medium was removed and replaced with fresh medium containing the same concentrations of DAMPs in the presence of 10% FCS as a cholesterol acceptor. BODIPY-associated fluorescence was measured in culture supernatants at the indicated time points, as described (31).

### Animal work

All procedures were carried out under a Home Office project license. Inbred 7 to 9-wk-old wild-type C57/BL6J mice were obtained from Charles River. Mice (n=5/group) were intraperitoneally injected with PBS (500 μl) or Aristolochic Acid (AA, Sigma, 2.5mg/kg), in the presence or absence of sTLR2 (12.5 µg/kg) or Paquinimod (1mg/kg). Injections were repeated every 3 days for a total of 4 times and mice were sacrificed 1 day (Day 10) or 10 days (Day 21) after the last injection. Blood was obtained by cardiac puncture and analysed by flow cytometry as described above. Following centrifugation, plasma was kept frozen (-80°C) until analysis by ELISA (cytokines and DAMP levels) or for creatinine levels (Cardiff and Vale UHB, Medical Biochemistry services). RNA was extracted as described above from the total blood cell fraction. The heart was flushed with PBS (10 ml) prior to aorta and kidney isolation. The thoracic portion of the aorta was immediately snap-frozen prior to RNA extraction and the right kidney was halved lengthwise, transferred to a histology cassette, and fixed in 10% Neutral-Buffered Formalin (24h). Cassettes were then transferred to 70% ethanol and kept at 4°C prior to embedding, sectioning (8 µm) and Masson’s trichrome staining (Bioimaging Hub, Cardiff University). For bacterial clearance experiments, Day 21-AAN mice were i.p injected with live *S.epidermidis* (5x10^8^ cfu/mouse), in the presence or absence of sTLR2 (12.5µg/kg). Peritoneal lavages and blood were obtained at 1h, 4h and 24 and bacterial numbers determined by colony counting 48h after spreading on agar plates.

### Statistical analysis

Statistical analyses are described in the corresponding figure legends. For experiments where data was not normally distributed, *p* values were calculated using a Mann-Whitney U test for independent samples or the non-parametric Wilcoxon signed-rank test for paired samples. In all other cases, shown *p* values were calculated using an unpaired Student *t*-test.

## Results

### A multi-TLR inhibition strategy inhibits vascular inflammation and pro-atherosclerotic gene expression in nephropathic mice without impairing infection clearance

**Figure 1:**
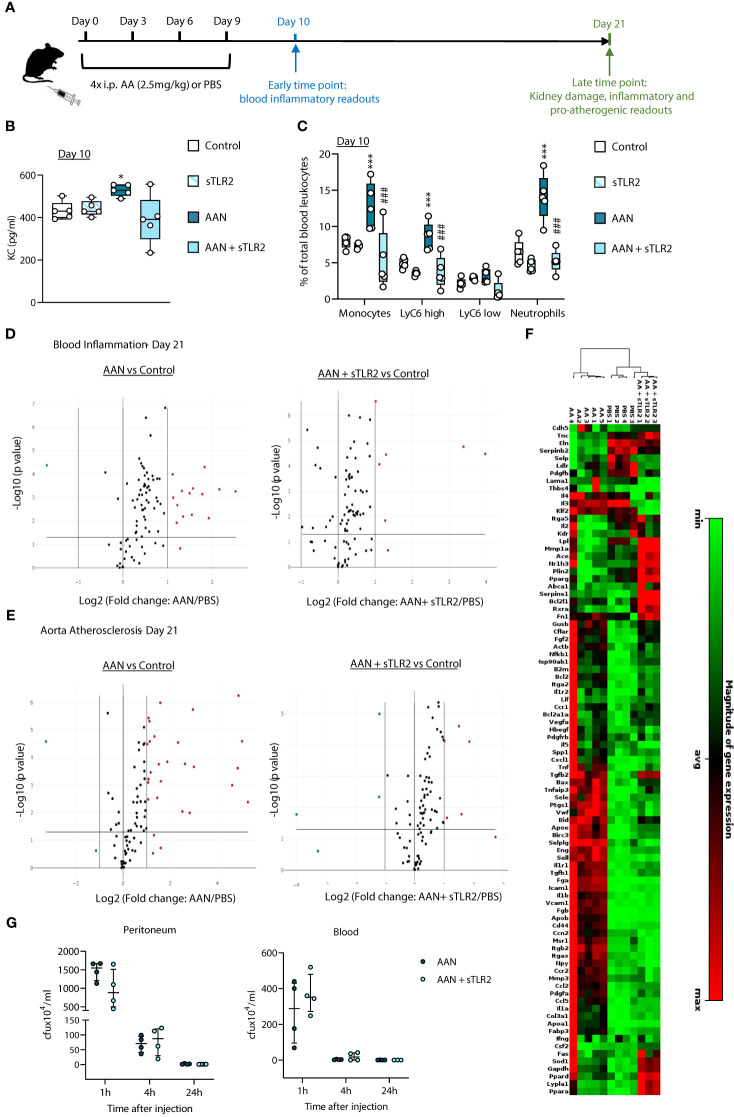
Soluble TLR2 administration inhibits AAN-induced systemic inflammatory and pro-atherosclerotic responses *in vivo.* C57BL/6J mice (n=5 per group) were injected intraperitoneally 4 times at 3-day intervals with AA (2.5 mg/kg) or PBS, in the presence or absence of sTLR2 (12.5µg/kg) **(A)**. Blood and aortas were obtained at Day 10 or Day 21, Day 0 being the day of the first injection. Cytokine plasma levels were determined by ELISA **(B)** and innate leukocyte proportions by flow cytometry (**C**, percentage of gated single cells shown). Open circles denote individual animals, horizontal bars indicate the median value. */#, *p*<0.05; ***/###, *p*<0.005 (*, AAN vs PBS*;* #, AAN +sTLR2 vs AAN), Mann-Whitney U test. The changes between AAN+ Paquinimod and Control groups were not significant. Volcano plots **(D, E)** compare the effect of AAN and AAN + sTLR2 on blood inflammation and immune responses-associated **(D)** or aortic atherosclerosis-associated **(E)** gene expression at Day 21. Red (upregulated, fold change ≥ 2) and green (downregulated, fold change ≤ 0.5) circles represent single genes significantly affected (*p* value < 0.05, represented by the horizontal line) compared to PBS control. Heatmap **(F)** displays experimental group hierarchical clustering, as determined according to the aortic expression levels of the 84 genes tested. Each column represents a sample; each row represents a gene; the relative gene expression scale is depicted on the right. At Day 21 **(G)** AAN mice were i.p injected with live *S.epidermidis* (5x10^8^ cfu/mouse), in the presence or absence of sTLR2 (12.5µg/kg). Peritoneal lavages and blood were obtained at the indicated time points and bacterial numbers determined by colony counting after growth.

Given the prior suggestion that TLR DAMPs may contribute to CV risk in CKD (25–27), we assessed the extent of their overall contribution to chronic vascular inflammation in nephropathic animals. A previously described mouse model of chronic Aristolochic Acid-induced Nephropathy (AAN) (32) was used (**Figure 1A**). Repeated administration of AA induced tubular injury leading to inflammation followed by tissue remodeling and fibrosis, as well as increased creatinine plasma levels [**Supplementary Figure 1** and (32)]. Of note, these effects were present at Day 21 and remained significant at Day 56, confirming the maintenance of kidney pathology (**Supplementary Figures 1B, C**).

AAN induced an early increase (Day 10) in plasma levels of the neutrophil chemoattractant KC, (**Figure 1B**) and markedly higher neutrophil (CD11b^+^/Ly6G^+^) and monocyte (CD11b^+^/Ly6G^-^) proportions (**Figure 1C**). Notably, the increase in total monocytes was mostly driven by the pro-inflammatory Ly6C^high^ subset (33), preferentially recruited to the atherosclerotic plaque (34) and specifically associated with increased risk of CV events in CKD (35).

To assess the overall contribution of TLR DAMPs to these chronic nephropathy-induced responses, a multi-TLR inhibition strategy was used. Soluble TLR2 (sTLR2) is a natural TLR inhibitor with anti-inflammatory capacity (36–40). It inhibits TLR activation by i) acting as a decoy receptor, preventing TLR2 ligand recognition by membrane TLR2, and ii) binding to and blocking the common TLR coreceptor CD14 (37). By targeting CD14, sTLR2 can inhibit the activation of CD14-dependent TLRs other than TLR2, notably TLR4 (38–40). sTLR2 administration reduced the AAN-induced effects on KC, neutrophils and Ly6C^high^ monocytes back to levels similar to PBS-injected mice (**Figures 1B, C**).

To gain a better insight into the range of lasting systemic inflammatory changes induced by AAN and the efficacy of multi-TLR inhibition as an anti-chronic inflammation strategy, blood expression of 84 inflammation and immunity-associated genes was investigated at the later time point, Day 21. Fourteen genes were upregulated by AAN, including those coding for acute phase proteins (Serum amyloid P, CRP) and pro-inflammatory mediators (Colony stimulating factor 2; IFN2α; IL-2; IL-6; IL-17α; IL-23α, Myeloperoxidase). Upregulated transcripts were also associated with the adaptive immune response: FOXP3, IL-2, IL-17, IL-23 and IL-5 (**Figure 1D**, **Supplementary Table 1**). Targeting the DAMP-TLR pathway for inhibition had a significant effect, as sTLR2 reduced to normal levels the expression of over half the genes, (**Figure 1D**, **Supplementary Table 1**), including that for the CV event predictor CRP.

In addition to inducing systemic inflammation, AAN led to a significant upregulation of pro-inflammatory and atherosclerotic-promoting genes in the aorta at Day 21. 26 out of 84 genes tested were found upregulated at this time point (**Figure 1E**, **Supplementary Table 1**), the majority involved in inflammation, endothelial activation and leukocyte recruitment to inflammatory sites, notably the atheroma, (*Ccl2, Ccl5, Ccr2, Cd44, Cxcl1, Fga, Fgb, Icam1, Il1a, Il1b, Itgax*, *Sele, Sell, Selplg, Tnf, Tnfaip3, Vcam1*), apoptosis (*Birc3*) and platelet activation (*Ptgs1*). Some affected genes were specific of the atherosclerotic process, notably those involved in lipid metabolism and handling (*Apoa1, Apob, Msr1*). sTLR2 markedly reduced the aortic expression of 25 out of the 26 AAN-induced atherosclerosis-associated genes: 20 were kept at normal levels, while 5 were reduced but remained elevated compared to the control (**Figure 1E**, **Supplementary Table 2**). Clustering analysis revealed that the aortic inflammatory and atherosclerosis-associated gene expression profile of AAN mice administered with sTLR2 was significantly closer to that of control than to AAN mice (**Figure 1F**).

Thus, sTLR2 effectively reduced AAN-induced key mediators of chronic vascular inflammation and pathology, demonstrating the extensive contribution of the DAMP-TLR pathway to these processes. Critically, sTLR2’s anti-inflammatory effect did not compromise the ability of AAN mice to clear a live bacterial infection, either at infection site (peritoneum) or escaped pathogens in the blood (**Figure 1G**).

### Calprotectin, Hsp70, HA and HMGB-1 are elevated in plasma from CKD patients

**Figure 2:**
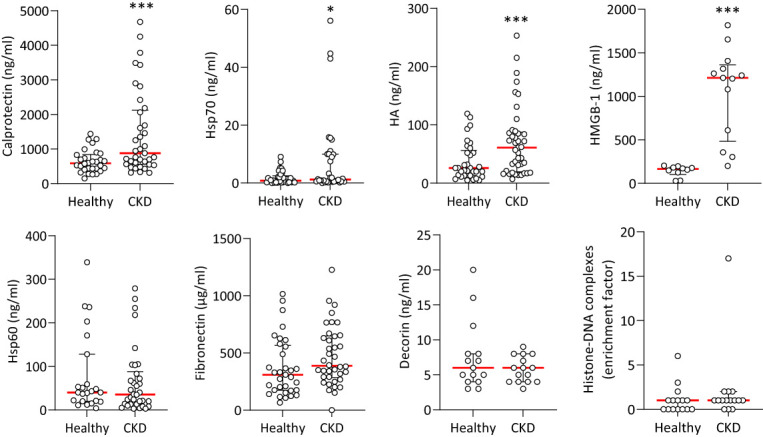
Plasma levels of TLR DAMPs in healthy individuals and CKD patients. Concentrations of TLR DAMPs in plasma from healthy donors and Stage 5 CKD patients. Horizontal red bars denote the median value, open circles denote individual donors. Hsp70, Hsp60, HA, Calprotectin, Fibronectin: Healthy n=30, CKD n=35, CKD + CVD n=38; Decorin: Healthy n=20, CKD n=25. HMGB-1, Histone-DNA complexes: Healthy n=12, CKD n=15. *, *p*<0.05; ***, *p*<0.005 (CKD vs Healthy), Mann-Whitney U test.

Given the extensive inhibitory effect of sTLR2 on nephropathy-induced vascular pathology observed here, the involvement of specific TLR2 and/or TLR4 DAMP agonists were investigated. We compared the levels of a number of known TLR DAMPs in plasma samples from stage 5 CKD patients with those from age-matched healthy donors (**Table 1**). Hsp60, Hsp70, HA, HMGB-1, Fibronectin, Decorin (TLR2 and TLR4 ligands), Calprotectin (S100A8/S100A9, TLR4 ligand), Histone H3 (TLR2 ligand) and Histone-DNA complexes (TLR2, TLR4 and TLR9 ligand) were selected to encompass well-described DAMPs involved in inflammatory pathologies (15). Histone H3 levels were below the detection threshold in both groups. Notably, Calprotectin, Hsp70, HA and HMGB-1 were elevated in patients’ plasma compared to controls (**Figure 2**).

To explore the range and extent of the pro-inflammatory and CV effects of the CKD-elevated DAMPs, their ability to promote key cellular functions critical to systemic and vascular chronic inflammation, endothelial dysfunction and atherosclerosis development was evaluated. First, it was confirmed that the purified DAMP preparations were not contaminated with significant levels of endotoxin. Specifically, only Hsp70 increased IL-8 production by human monocytes, and this increase was not inhibited by the LPS antagonist Polymyxin B (**Supplementary Figure 2A**) but was abrogated following denaturation by boiling (**Supplementary Figure 2B**), which does not affect endotoxin. Our LAL measurements confirmed the very low endotoxin content of the DAMP preparations (**Supplementary Figure 2C**).

### CKD-associated DAMPs promote endothelial dysfunction-associated responses

**Figure 3:**
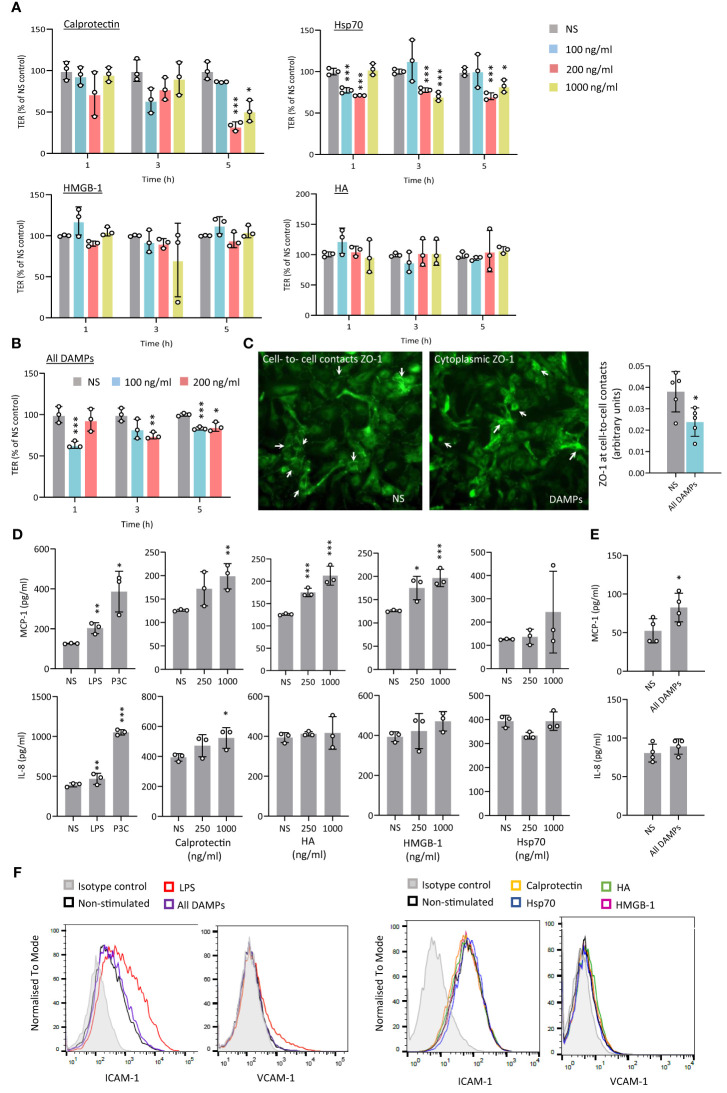
CKD-associated DAMPs decrease transendothelial resistance and induce limited pro-inflammatory responses by human arterial endothelial cells. **(A-C)** Triplicate cultures of human umbilical arterial endothelial cells were grown to confluence prior to stimulation or not (NS) with single **(A)** or combined CKD-associated DAMPs **(B, C)** at the indicated concentrations **(A, B)** or 100 ng/ml **(C)**. Trans-endothelial electrical resistance (TER) was measured at the indicated time points **(A, B)** and ZO-1 expression **(C)** was quantified at cell-to-cell contacts 5h following DAMP stimulation. White arrows in **(C)** indicate preferential ZO-1 localisation at cell-to-cell in resting conditions (NS) or in the cytoplasm following DAMP treatment. Individual data points from 3 independent experiments, (mean +/- SD) are shown **(A, B)** or 1 experiment representative of 3 **(C)** *, *p*<0.05; **, *p*<0.01; ***, *p*<0.005, Stimulation vs no stimulation, unpaired Student’s *t* test. D-F. Triplicate cultures of human aortic endothelial cells were stimulated (18h) with LPS (10 ng/ml), Pam_3_CSK_4_ (P3C, 500 ng/ml) or the indicated concentrations of Calprotectin, Hsp70, HMGB-1, or HA, alone **(D, F)** or combined (**E, F**, 1 µg/ml). Cytokine levels in culture supernatants **(D, E)** are shown as individual data points from one experiment representative of 4 (mean +/- SD). *, *p*<0.05; **, *p*<0.01; ***, *p*<0.005, Stimulation vs no stimulation, unpaired Student’s *t* test. ICAM-1 and VCAM-1 surface expression levels are shown (**F**, 20,000 cells/condition), from 1 experiment representative of 3.

Increased permeability of the endothelial barrier promotes local inflammation and atherosclerosis development and occurs at a very early stage of the disease (41, 42). Endothelial cell exposure to Calprotectin, Hsp70, or a combination of the 4 CKD-DAMPs, but not to HA or HMGB-1, resulted in a significant drop in trans-endothelial resistance (TER, **Figures 3A, B**). In line with the drop in TER, DAMP treatment resulted in decreased cell-to-cell contact localisation of Zonula occludens-1 (ZO-1), a key scaffold protein involved in tight junctions (29) (**Figure 3C**). This effect was not associated with a decrease in cell numbers or coverage (**Supplementary Figure 3**).

In vascular inflammation, endothelial cell activation initiates monocyte recruitment to the intima, notably via endothelial production of chemoattractants. Exposure of Human Aortic Endothelial Cells (HAEC) to Calprotectin, HA and HMGB-1, as well as all CKD-DAMPs combined, but not to Hsp70, led to a modest increase in MCP-1, a major monocyte chemoattractant expressed at atherosclerosis plaques (**Figures 3D, E**). In addition, Calprotectin, but not the other DAMPs, induced a modest release of the neutrophil chemoattractant IL-8 (**Figure 3D**). HAEC showed, however, high sensitivity to TLR stimulation with bacterial components (**Figure 3D**). HAEC co-stimulation with oxidized low-density lipoprotein (OxLDL), a DAMP and potent pro-atherogenic lipid recognised by TLR2 and TLR4 at the plaque (12), did not alter the DAMPs’ response profile (**Supplementary Figure 4**). Endothelial expression of adhesion molecules such as Intercellular Adhesion Molecule-1 (ICAM-1) and Vascular Cell Adhesion Molecule-1 (VCAM-1) also controls monocyte recruitment to the intima. Their expression increased following HAEC exposure to LPS, but not to individual or combined CKD-DAMPs (**Figure 3F**). Similarly, Tissue Factor (TF) production by endothelial cells, which contributes to CV events by driving coagulation (43), was undetectable, irrespective of exposure to CKD-DAMPs, either with or without OxLDL.

Thus, while only inducing modest inflammatory responses by endothelial cells, Calprotectin and Hsp70, but not HA or HMGB-1, showed the capacity to disturb the endothelial barrier, a first step towards atherosclerosis, PVD and CAD.

### CKD-associated DAMPs promote monocyte chemotaxis

**Figure 4:**
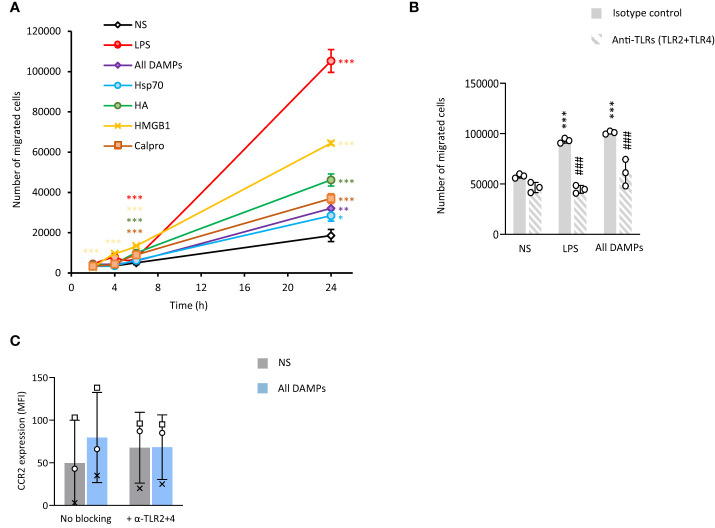
CKD-associated DAMPs increase the migratory capacity of monocytes towards MCP-1 in a TLR-dependent manner. Triplicate cultures of Mono-Mac 6 monocytes were stimulated (18h, 37°C) with LPS (10 ng/ml) or the indicated DAMPs (1 µg/ml), alone or in combination after pre-exposure (1h, 37°C, **B, C**) or not **(A)** to a combination of anti-TLR2 and anti-TLR4 blocking antibodies (5 µg/ml) or the relevant isotype control (10 µg/ml). **(A, B)** Cells were subsequently starved in serum-free medium for 1 h prior to seeding (200,000 cells, in triplicates) in the top chamber of 8 µm pores trans-wells. The bottom compartment was filled with RPMI + 10% serum + MCP-1 (50 ng/ml). Cell numbers were counted in the bottom compartment after 2h, 4h, 6h **(A)** and 24h **(A, B)**. Results shown are the mean +/- SD from one experiment representative of 7 (A, LPS and all DAMPs combined) or 3 (**A**, single DAMPs), or from 3 experiments (**B**, open circles denote individual experiments). *, *p*<0.05; **, *p*<0.01; ***, *p*<0.005; *, Stimulation vs no stimulation or ^#^, anti-TLR antibodies vs isotype, unpaired Student’s *t* test **(A)**, paired Student’s *t* test **(B)**. Cells were then analysed by flow cytometry for the levels of CCR2 **(C)**. Results are shown as mean fluorescence intensity (MFI) for 20,000 cells/condition from 3 independent experiments. Identical symbols identify paired results. *, *p*<0.05, All DAMPs vs no stimulation (NS), paired t-test.

Monocyte recruitment to the intima during vascular inflammation is largely mediated by MCP-1 (44, 45). Monocyte exposure to all 4 identified CKD-DAMPs, in combination or alone, increased their migration towards MCP-1 (**Figure 4A**). While these DAMPs all have immune receptors other than TLRs, blocking TLR2 and TLR4 inhibited most of this effect, indicating their major role in the DAMPs-induced migration (**Figure 4B**). Mechanistically, monocyte expression of CD11b, integrin α4 and PSGL-1, adhesion molecules involved in cell extravasation into the intima, was not affected by exposure to the CKD-DAMPs (**Supplementary Figure 5**). The DAMPs, however, upregulated monocyte expression of the MCP-1 receptor CCR2, in a TLR2/4-dependent manner (**Figure 4C**).

### CKD-associated DAMPs promote pro-inflammatory mediators production and foam cell formation by macrophages

**Figure 5:**
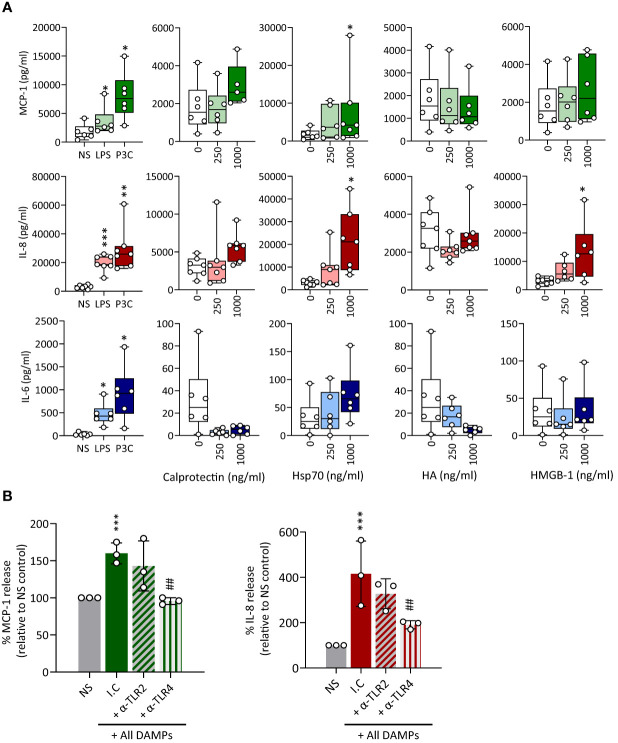
CKD-associated DAMPs induce pro-inflammatory mediator production in a TLR-dependent manner. **(A)** Triplicate cultures of primary monocyte M-CSF-derived macrophages were stimulated (18h, 37°C) with LPS (10 ng/ml), Pam_3_CSK_4_ (P3C, 500 ng/ml) or the indicated concentrations of Calprotectin, Hsp70, HMGB-1, or HA. **(B)** Triplicate cultures of macrophages were pre-exposed (1h, 37°C) to anti-TLR2 or anti-TLR4 blocking antibodies (10 µg/ml) or the relevant isotype control (I.C), prior to stimulation (18h, 37°C) with all DAMPs combined (each at 1 µg/ml). Levels of the indicated cytokines in culture supernatants were determined by ELISA. Results shown in **(A)** are from at least 6 experiments performed with cells from different donors, each represented by an open circle. Horizontal lines in boxes denote the median value. **(B)** shows the average cytokine production relative to control for 3 experiments, each depicted by an open circle. */#, *p* <0.05; **/##, *p* <0.01; ***/###, *p* <0.005, **(A)** Stimulation vs no stimulation, Wilcoxon signed-rank test (paired samples), **(B)** *, Stimulation vs no stimulation, ^#^, αTLR antibody vs isotype control (I.C.), unpaired Student’s *t* test.

Production of key pro-inflammatory mediators, such as MCP-1, IL-8 and IL-6, by recruited plaque monocyte-derived macrophages is a key component of the vascular inflammation observed in atherosclerosis and CAD (34, 46–48). Hsp70 stimulated the release of all 3 mediators tested and Calprotectin only that of MCP-1 and IL-8, although its effect was not statistically significant (**Figure 5A**). HMGB-1 induced only the release of IL-8. Of note, Calprotectin and HA appeared to reduce IL-6 release, albeit non-significantly. Blocking TLR2 and TLR4 showed that TLR4 mediated most of the DAMPs-induced pro-inflammatory mediator release (**Figure 5B**).

**Figure 6:**
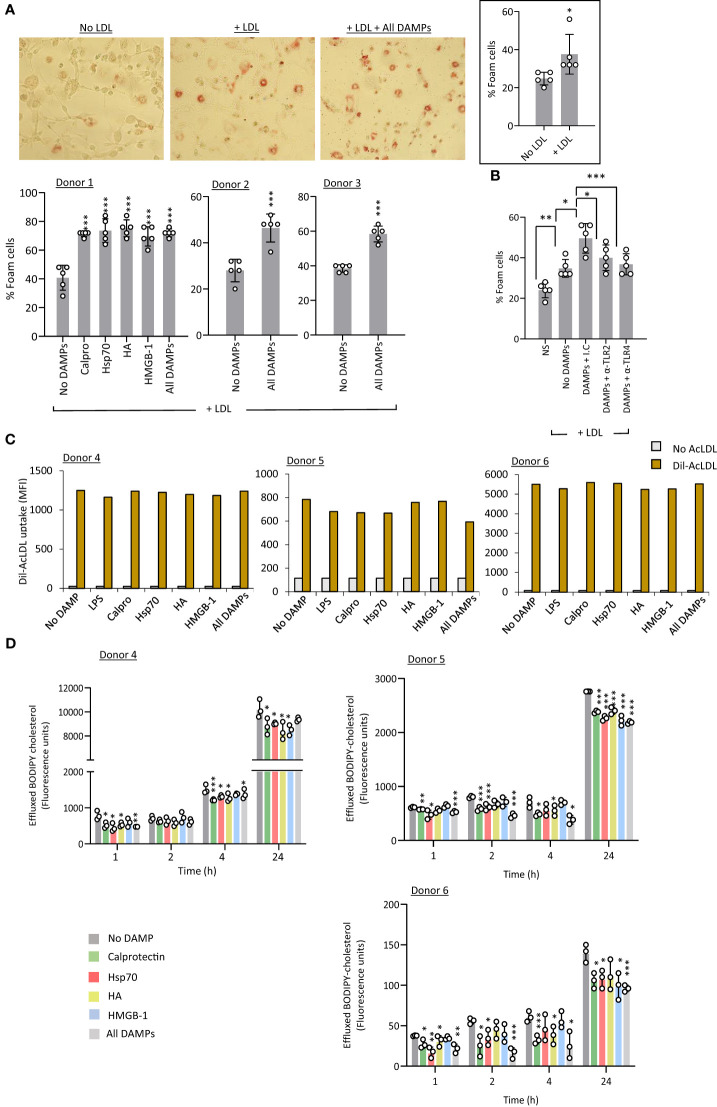
CKD-associated DAMPs promote foam cell formation in a TLR-dependent manner by reducing cholesterol efflux. **(A, B)** M-CSF-differentiated macrophages were exposed (24h) to LDL (25 µg/ml) in the presence or absence of the indicated DAMPs, alone or in combination (1 µg/ml) and of the indicated anti-TLR blocking antibodies or relevant isotype control (10 µg/ml, **B**) prior to staining with Oil Red-O for lipid visualisation by light microscopy (representative images shown). Plots show the percentage of foam cells in each condition. **(C)** M-CSF-differentiated macrophages were starved in medium supplemented with 0.2% fatty-acid free BSA (18h) in the presence or absence or the indicated DAMPs, alone or in combination (1 µg/ml) before addition of Dil-conjugated acetylated LDL (Dil-AcLDL, 10 µg/ml). After 24h, internalised Dil-AcLDL was quantified by flow cytometry (20,000 events/condition, MFI shown) **(B)** Triplicate cultures of M-CSF-differentiated macrophages were loaded with BODIPY-labelled cholesterol (5 µM, 18h), before medium removal and exposure to the indicated DAMPs, alone or in combination (1 µg/ml). After equilibration (1h), medium was removed and replaced with fresh medium containing the same concentrations of DAMPs in the presence of 10% FCS as a cholesterol acceptor. BODIPY-associated fluorescence was measured in culture supernatants at the indicated time points. Results are mean +/- SD obtained with macrophages prepared from 3 different donors (each donor shown, **A, D**) or representative of 3 donors tested **(B)**, or MFI from 20,000 macrophages from 3 donors tested (**C**, each donor shown). Open circles show individual data points for each mean (**A, B**, 5 individual fields of view per experimental group, **(D)**, individual triplicates) *, *p*<0.05; **, *p*<0.01; ***, *p*<0.005; *DAMP stimulation vs no DAMP **(A, B, D)** or as indicated **(C)**, unpaired Student’s *t* test.

Foam cells are lipid-laden macrophages unique to the atherosclerotic plaque which drive disease progression. Macrophage-to-foam cell transition is a hallmark of the atherosclerosis process (45). Therefore, a potential effect of CKD-DAMPs on foam cell formation was investigated. Exposure to LDL alone resulted in a modest rise in foam cell numbers (**Figure 6A**, inset), but co-treatment with the 4 individual CKD-DAMPs or all CKD-DAMPs combined further increased foam cell formation (**Figure 6A**). TLR4 and TLR2 both mediated the DAMPs’ effect (**Figure 6B**).

Foam cell formation may result from excessive uptake and/or reduced cholesterol efflux. Exposure to single or combined CKD-DAMPs did not affect macrophage uptake of fluorescent-modified LDL (**Figure 6C**). However, (BODIPY) cholesterol efflux was significantly reduced following macrophage exposure to the CKD-associated DAMPs, either alone or in combination, following cholesterol loading (**Figure 6D**).

Thus, all identified CKD-DAMPs, alone or in combination, can promote foam cell formation in a TLR2/4-dependent manner and this effect likely results from the DAMPs’ ability to reduce cholesterol efflux. Of note, we verified that uremic conditions (cells cultured in uremic patient serum) did not affect the ability of cells to respond to DAMP stimulation, as judged by monocyte migration, pro-inflammatory mediators’ production, and foam cell formation by macrophages (**Supplementary Figure 6**)

Together, the *in vitro* findings demonstrated the different potential of the 4 identified CKD-DAMPs to promote typical pro-inflammatory functions by cell types critical to vascular chronic inflammation and CV pathology. Although immune receptors other than TLRs have been reported for the 4 CKD-DAMPs tested, TLR2/4 appear as the main mediators of DAMPs-induced responses, consistent with the inhibitory effect of sTLR2 on vascular inflammation in AAN mice.

### Single DAMP blockade efficiently reduces vascular inflammation and pro-atherosclerotic gene expression in nephropathic mice

**Figure 7:**
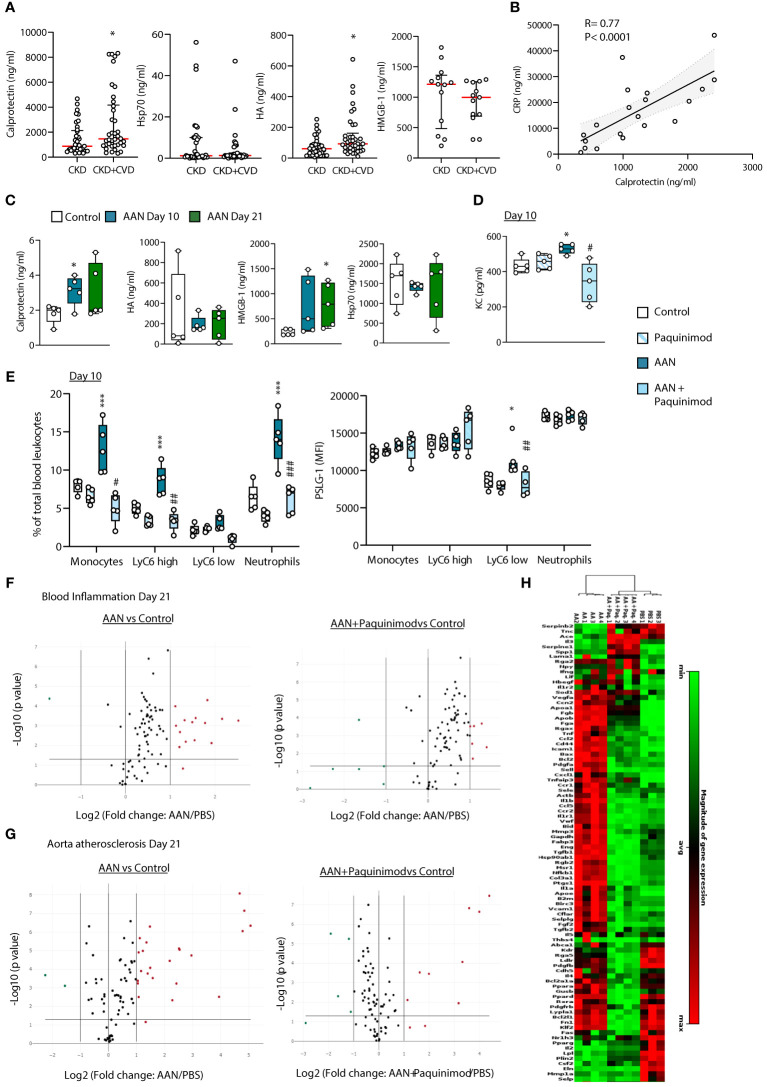
Calprotectin blocking inhibits AAN-induced systemic inflammatory and pro-atherosclerotic responses *in vivo.* **(A)** Concentrations of TLR DAMPs in plasma from Stage 5 CKD patients without prior CVD diagnosis or CKD patients with prior CVD diagnosis **(B)**. Horizontal red bars denote the median value, open circles denote individual donors. *, *p*<0.05 (CKD+CVD vs CKD), Mann-Whitney U test. **(B)** Correlation between plasma levels of Calprotectin and CRP or HA and CRP in CKD+CVD patients. Statistical analysis was done using a Spearman’s rank correlation test. **(C–H)**. C57BL/6J mice (n=5 per group) were injected intraperitoneally 4 times at 3 days intervals with AA (2.5 mg/kg) or PBS, in the presence or absence of Paquinimod (1 mg/kg). Blood and aortas were obtained at Day 10 (blood only) or Day 21, Day 0 being the day of the first injection. DAMP **(C)** and cytokine **(D)** plasma levels were determined by ELISA, and innate leukocyte proportions (percentage of gated single cells shown) and PSLG-1 expression levels **(E)** by flow cytometry. Circles denote individual animals, horizontal bars indicate the median value. */#, *p*<0.05; **/##, *p*<0.01; ***, *p*<0.005 (*, AAN vs PBS*;* #, AAN +Paquinimod vs AAN), Mann-Whitney U test. The changes between AAN+ Paquinimod and Control groups were not significant. Volcano plots **(F, G)** compare the effect of AAN and AAN + Paquinimod on inflammation and immune responses in the blood **(F)** or atherosclerosis-associated gene expression in aortas **(G)** at Day 21. Red (upregulated, fold change ≥ 2) and green (downregulated, fold change ≤ 0.5) circles represent single genes significantly affected (*p* value < 0.05, represented by the horizontal line) compared to PBS control. Heatmap in **(H)** displays experimental group hierarchical clustering, as determined according to the aortic expression levels of the 84 genes tested. Each column represents a sample; each row represents a gene; the relative gene expression scale is depicted on the right.

We next investigated whether direct DAMP inhibition may provide an alternative approach to multi-TLR inhibition to reduce CKD-associated vascular pathology. To select a single TLR DAMP for targeting, plasma levels of the 4 CKD-associated DAMPs were compared between CKD patients who had not and those who had been diagnosed with CVD at the time of sampling (the latter excluded from the analysis in **Figure 2**). Analysis was limited to CVD diagnosis directly related to arteriosclerosis, namely ischemic heart disease and peripheral vascular disease (**Table 1**). The occurrence of CVD in CKD was associated with higher plasma levels of Calprotectin and HA, but not HMGB-1 or Hsp70 (**Figure 7A**), suggesting potential causal relationship. Stage 5 CKD patients with diagnosed CVD were significantly older than those without (**Table 1**). However, neither Calprotectin nor HA levels correlated with age in either cohort (**Supplementary Figure 7**), suggesting that age is not driving the association between elevated DAMP levels and CVD. In Stage 5 CKD patients with prior CV diagnosis, levels of Calprotectin, but not HMGB-1, HA or Hsp70 (**Supplementary Figure 8**), correlated strongly with CRP, a well-established predictor of CV events (5, 49–51) (**Figure 7B**). Together with the effects of Calprotectin on vascular responses *in vitro*, these observations suggested that Calprotectin may play a critical role in chronic vascular inflammation and increased CV risk in CKD and thus was selected for specific inhibition *in vivo*.

In AAN mice, Calprotectin, as well HMGB-1, but not Hsp70 or HA, was found elevated (**Figure 7C**), and its pharmacological inhibition with Paquinimod (ABR-215757), which interacts specifically with the S100A9 subunit and blocks its recognition by TLR4 (52, 53), prevented the AAN-induced increase in plasma KC (**Figure 7D**) and innate immune cell proportions (total and Ly6C^high^ monocytes as well as neutrophils, **Figure 7E**). Ly6C^low^ monocytes from AAN mice displayed increased expression of PSGL-1, which mediates monocyte adhesion to the endothelium during recruitment to form the atherosclerotic plaque (44) and this increase was prevented by Paquinimod (**Figure 7E**). Calprotectin blockade also reduced the expression of 10 out of 14 of the AAN-upregulated immune and inflammatory genes in blood cells at Day 21 back to normal levels (**Figure 7F**, **Supplementary Table 3**) and robustly inhibited the AAN-induced aortic overexpression of most pro-atherosclerotic genes tested (**Figure 7G**, **Supplementary Table 4**). As described for sTLR2, the overall inflammatory and atherosclerosis-associated aortic gene expression cluster heat map of AAN mice treated with Paquinimod was significantly closer to that of controls than to AAN mice (**Figure 7H**).

Thus, pharmacological inhibition of a single DAMP, notably Calprotectin, provides another efficient therapeutic approach to reduce vascular pathology in CKD.

## Discussion

CKD is associated with markedly increased cardiovascular risk (4, 54, 55). Established CVD treatments for non-CKD patients are less effective in CKD (54) and suboptimal responses to therapies are believed to be underpinned by the unique state of chronic inflammation in CKD. Therefore, better knowledge of the mechanisms driving chronic and vascular inflammation in CKD is required before effective treatments for CV diseases can be developed.

While DAMPs’ involvement in CV risk in CKD has been suggested, the extent of their effect, their comparative individual contribution to CKD-associated vascular pathology, and the evaluation of potential therapeutic strategies, have remained largely undescribed. Here, we evaluated in nephropathic mice two potential TLR DAMP-targeting therapeutic strategies to prevent or lower vascular inflammation and pro-atherosclerotic responses, thereby demonstrating the critical role of the DAMP-TLR pathway in the process and highlighting potential single DAMP targets. We also described the different abilities of four CKD-associated DAMPs to promote key cellular responses associated with vascular inflammation and worsening of atherosclerosis.

The extent of TLR DAMPs’ contribution to vascular inflammation and pro-atherosclerotic responses in CKD was revealed by the near complete blockade of the vascular inflammatory readouts tested following administration of the multi-TLR inhibitor sTLR2 in nephropathic mice. Although most DAMPs have immune receptors other than TLRs, this finding indicates a critical involvement of TLRs in mediating further vascular inflammation in CKD and is the first report of the efficiency of this approach in CKD-associated pathology. Of note, sTLR2 did not reduce kidney damage in this model, as judged by the maintenance of fibrosis levels (**Supplementary Figure 9**), suggesting that its systemic anti-inflammatory effect was mediated by direct targeting of chronic vascular responses to DAMPs, rather than by indirect inhibition of kidney damage.

Mechanistically, the 4 identified CKD-DAMPs differentially promoted several key cellular responses associated with systemic and vascular inflammation and atherosclerosis development. These activities were largely TLR-dependent, consistent with the effect of sTLR2 *in vivo*. The responses to these DAMPs could not be anticipated, as different TLR ligands may induce qualitatively and quantitatively different responses (56–58), and different DAMPs may involve different TLR coreceptors, and have other non-TLR immune receptors (59–61). Accordingly, different DAMPs showed varying effects or no effect, suggesting the engagement of different signalling pathways and that specific DAMPs may be more pathological than others and thus may be more promising therapeutic targets.

One limitation of the *in vitro* experiments is that different endothelial cell lines were used to evaluate the effect of CKD-DAMPs on endothelial cell responses. Our endothelial cells of choice, the HAECs, could not be grown to form a fully confluent single layer on polyester trans-well insets, a critical step to TER measurements. Therefore, we selected the human umbilical arterial endothelial cells, rather than human umbilical veinous endothelial cells, for these experiments, as their arterial origin is more relevant to the study of atherosclerosis-associated responses.

Of the 4 CKD-DAMPs tested, Calprotectin was selected as a candidate for direct therapeutic blockade, as it consistently affected the pro-atherosclerotic functions tested *in vitro*, was further elevated in CVD-diagnosed CKD patients and highly correlated with the predictor of CV events CRP, suggesting a potential causal relationship between high Calprotectin levels and CVD in CKD patients. Calprotectin was also elevated in AAN mice and its pharmacological blockade abrogated chronic nephropathy-induced systemic chronic inflammation and robustly reduced vascular inflammatory and pro-atherosclerotic gene expression, indicating a key role for Calprotectin in mediating these activities. While elevated plasma levels of Calprotectin, as well as of other DAMPs, have been shown to predict CV mortality in the non-CKD population (19), this is the first description of the role of Calprotectin in mediating CV risk in the CKD population and of its potential as a therapeutic target against vascular inflammation leading to CVD. Interestingly, overexpression of S100A12, a Calprotectin family member, induced cardiac damage and dysfunction in CKD animals, although in a RAGE-mediated manner (27).

The fact that both sTLR2 and Paquinimod were able to inhibit long-term AAN-induced changes while only being administered during CKD induction suggests that DAMPs contribution to CV pathology in CKD starts early during the CKD process. While the beneficial effect of early DAMP inhibition appeared to be maintained for some time in this model, probably by delaying the systemic and vascular inflammatory consequences of AAN, it is likely that anti-inflammatory therapeutics would need to be routinely administered to patients with ongoing CKD.

Damaged kidney tissue may contribute to elevated DAMP plasma levels and chronic inflammation in CKD, therefore, models of nephropathy by kidney removal were not appropriate for this study. Similarly, mouse models of hyperlipidemia-induced full-blown atherosclerosis (e.g., ApoE^-/-^ or LDLR^-/-^ mice on a high-fat diet) were not used here, since the lipid profiles in these animals is expected to be different from late-stage CKD patients, who in addition often develop atherosclerosis without hyperlipidemia, as opposed to the general population and in line with the lack of statin protection in these patients. Therefore, to reproduce atherosclerosis in CKD, a mouse model combining kidney injury, uremia and mild-moderate, but not severe, hyperlipidemia, would need to be developed, which was beyond the scope of this project. While, the AAN mice did not develop full-blown atherosclerosis, however signs of ongoing dysregulated vascular inflammation were apparent, which is the starting point of a number of CV pathologies, such as PVD or CAD. It is reasonable, therefore, to expect that plaque burden would be reduced by maintaining reduced vascular inflammation and arterial atherosclerosis-associated gene expression through the therapeutic strategies tested here.

**Figure 8:**
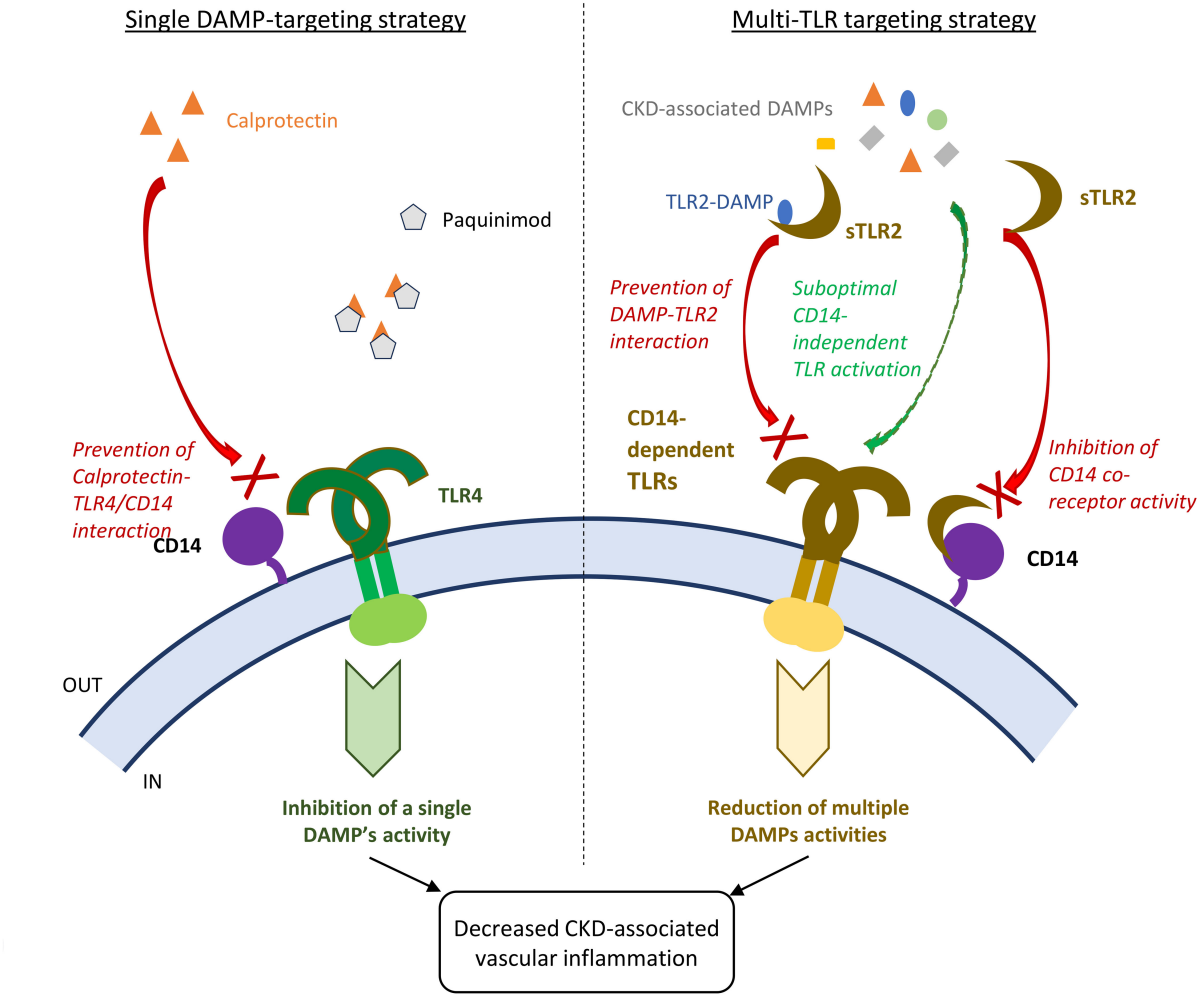
Schematic comparison of single DAMP and multiple TLR-targeting strategies to reduce vascular inflammation and disease in CKD patients. Single CKD-associated DAMP targeting strategy: for example, Calprotectin blockade with the pharmacological inhibitor Paquinimod at optimal dose is expected to achieve robust inhibition of the single DAMP’s vascular pro-inflammatory activity. Multi-TLR blocking strategy: sTLR2 will provide partial inhibition of the activity of all DAMP ligands, except for TLR2 DAMP ligands, as inhibition will rely just on the ability of sTLR2 to interact with the common co-receptor CD14 and prevent its enhancing activity, while some CD14-independent suboptimal TLR activation may remain. In the case of TLR2 DAMPs, sTLR2 will prevent their interaction with TLR2 due to its decoy receptor activity, in addition to preventing co-receptor activity. This study demonstrated that both strategies for DAMP inhibition were efficient at lowering CKD associated vascular inflammation.

Recent trials using monoclonal antibodies to IL-1β demonstrated that reducing chronic inflammation effectively reduces CV risk, notably in CKD patients. However, this particular strategy was associated with an increased risk of severe infections leading to death (9, 62), highlighting the need for other targets and safer approaches, especially in populations prone to infections. Here, sTLR2 was selected over a combination of anti-TLR antibodies, as full TLR blockade may increase infection risk. By contrast, the sTLR2 anti-inflammatory strategy does not compromise bacterial clearance, as we previously demonstrated in normal mice (37) and here in nephropathic mice. Of note, the use of Paquinimod alone was not associated with reduced pathogen clearance in bacterial and fungal infections models (63, 64), while dual HMGB-1 and Calprotectin blockade was (63). Therefore, the 2 proposed strategies appear as promising alternatives to reduce chronic inflammation and CV risk effectively and safely (**Figure 8**). They may be differentially effective in different patients, while specific DAMP blockade may be particularly efficient in patients with high levels of a single DAMP, sTLR2 may be a better option in patients with moderate or high levels of several DAMPs. We observed that individual DAMP levels may be independently elevated in CKD patients, as no correlations were found between levels of Calprotectin, HA, HMGB-1 and Hsp70 (not shown). In cases however when a particular DAMP may be significantly more elevated than others, the efficacy of single inhibition of that DAMP may be tested first, due to the generally lower production cost and increased stability of pharmacologic inhibitors compared to biologics such as sTLR2.

In addition to CVD, chronic inflammation is thought to drive other pathologies in the CKD cluster, such as diabetes (65), Alzheimer’s disease (66) and rheumatoid arthritis (15). Therefore, targeting the DAMP-TLR pathway may help to better manage several multimorbidites in the CKD cluster simultaneously.

## Data availability statement

The datasets presented in this study can be found in online repositories. The names of the repository/repositories and accession number(s) can be found below: GSE236195 (GEO).

## Ethics statement

The studies involving humans were approved by the Wales Kidney Research Tissue Bank. The studies were conducted in accordance with the local legislation and institutional requirements. The participants provided their written informed consent to participate in this study. The animal study was approved by the Animal Welfare and Ethical Review Body (Cardiff University) and the UK Home Office. The study was conducted in accordance with the local legislation and institutional requirements.

## Author contributions

A-CR and MOL contributed to conception and design of the study. MM, EC and A-CR carried out the bulk of the experiments and analysis. MB, IM and NI supervised or carried out specific experiments/techniques. CPS, DPR and TRH provided specific expertise. MM, EC and A-CR prepared figures. A-CR and MOL wrote the first draft of the manuscript. All authors contributed to the article and approved the submitted version.
