# Supplementary material for: Therapeutic targeting of chronic kidney disease-associated DAMPs differentially contributing to vascular pathology
Source: Front Immunol. 2023 Oct 2;14:1240679. doi: 10.3389/fimmu.2023.1240679 (PMC10577224; doi:10.3389/fimmu.2023.1240679)
Supplement: Supplementary file 1 [file DataSheet_1.pdf]

Supplementary Material

Therapeutic targeting of Chronic Kidney Disease-associated DAMPs differentially contributing to vascular pathology.

Authors: Morgane Mazzarino,[$] Esra Cetin,[$], Maria Bartosova, Iva Marinovic, Natacha Ipseiz, Timothy Hughes, Claus P. Schmitt, Dipak P. Ramji , Mario O. Labéta[§] and Anne-Catherine Raby,[§]

[$,§] These authors contributed equally

Correspondence: Anne-Catherine Raby or Mario O Labéta, Division of Infection & Immunity, School of Medicine, Cardiff University, Tenovus Building, Heath Park, Cardiff, CF14 4XN, United Kingdom. E-mail: RabyA@cardiff.ac.uk, Labeta@cardiff.ac.uk; phone: +44(0)2920687324, +44(0)2920687019; fax: +44 (0)29206 87303.

Supplementary Table 1. Effect of sTLR2 on AAN-induced changes in inflammation and immunity gene expression in blood at Day 21

		AAN *		AAN + sTLR2	
**Gene symbol**	**Description**	**Fold Change****	***p* Value****	**Fold Change****	***p* Value****
Apcs	Serum amyloid P-component	**3.0**	*0.0003*	1.3	*0.0084*
Crp	C-reactive protein	**4.5**	*0.0004*	1.7	*0.222*
Csf2	Colony stimulating factor 2	**3.9**	*0.0072*	1.1	*0.8473*
Foxp3	Forkhead box P3	**2.4**	*0.0017*	1.8	*0.0041*
Ifna2	Interferon alpha 2	**2.9**	*0.0007*	-1.6	*0.0280*
Ifnb1	Interferon beta 1	**2.5**	*0.0004*	-1.3	*0.0904*
Il17a	Interleukin 17a	**3.5**	*0.0006*	-1.6	*0.1015*
Il2	Interleukin 2	**2.2**	*0.0009*	-1.2	*0.0256*
Il23a	Interleukin 23a	**2.3**	*0.0106*	2.7	*0.0003*
Il5	Interleukin 5	**3.6**	*0.0001*	2.5	*0.0303*
Il6	Interleukin 6	**3.0**	*0.0048*	2.6	*0.0001*
Mbl2	Mannose-binding lectin (protein C) 2	**5.8**	*0.0006*	2.9	*0.2093*
Mpo	Myeloperoxidase	**2.6**	*0.0061*	2.6	*0.0044*
Mx1	Myxovirus (influenza virus) resistance 1	**2.2**	*0.0001*	2.4	*0.0142*
Rag1	Recombination activating gene 1	**-3.2**	*0.0001*	-5.8	*0.0001*

*****Only statistically significant (p<0.05) AAN-induced ≤-2 (in green) or ≥ 2 (in red) fold changes were considered.

******Compared to PBS control group.

Supplementary Table 2. Effect of sTLR2 on AAN-induced changes in atherosclerosis-related aortic gene expression at Day 21.

		**AAN ***		**AAN + sTLR2**	
**Gene symbol**	**Description**	**Fold Change****	***p* Value****	**Fold Change****	***p* Value****
Apoa1	Apolipoprotein A-I	**39.3**	*0.0041*	3.6	*0.0001*
Apob	Apolipoprotein B	**29.1**	*0.0002*	1.7	*0.0138*
Bcl2	B-cell leukemia/lymphoma 2	**2.1**	*0.0033*	1.5	*0.0031*
Birc3	Baculoviral IAP repeat-containing 3	**2.2**	*0.0000*	1.3	*0.0011*
Ccl2	Chemokine (C-C motif) ligand 2	**3.0**	*0.0007*	1.5	*0.0116*
Ccl5	Chemokine (C-C motif) ligand 5	**2.7**	*0.0029*	1.2	*0.5196*
Ccr2	Chemokine (C-C motif) receptor 2	**2.3**	*0.0003*	1.1	*0.4255*
Cd44	CD44 antigen	**2.9**	*0.0002*	1.2	*0.0061*
Cxcl1	Chemokine (C-X-C motif) ligand 1	**7.1**	*0.0101*	1.9	*0.0305*
Fga	Fibrinogen alpha chain	**29.8**	*0.0001*	1.5	*0.0583*
Fgb	Fibrinogen beta chain	**33.1**	*0.0001*	2.0	*0.0001*
Icam1	Intercellular adhesion molecule 1	**3.0**	*0.0001*	1.1	*0.0101*
Il1a	Interleukin 1 alpha	**5.7**	*0.0090*	1.9	*0.0292*
Il1b	Interleukin 1 beta	**8.0**	*0.0002*	1.0	*0.9634*
Il1r2	Interleukin 1 receptor, type II	**2.5**	*0.0652*	2.1	*0.0213*
Itgax	Integrin alpha X	**3.7**	*0.0001*	-1.4	*0.0918*
Itgb2	Integrin beta 2	**2.0**	*0.0001*	-1.1	*0.1180*
Msr1	Macrophage scavenger receptor 1	**2.1**	*0.0006*	-1.3	*0.1892*
Npy	Neuropeptide Y	**6.2**	*0.0002*	-15.9	*0.0165*
Ptgs1	Prostaglandin-endoperoxide synthase 1	**2.1**	*0.0001*	1.1	*0.3530*
Sele	Selectin, endothelial cell	**2.1**	*0.0008*	1.2	*0.1149*
Sell	Selectin, lymphocyte	**7.8**	*0.0001*	-1.2	*0.5354*
Selplg	Selectin, platelet (p-selectin) ligand	**2.2**	*0.0001*	-1.2	*0.1958*
Tnf	Tumor necrosis factor	**24.8**	*0.0010*	6.7	*0.0874*
Tnfaip3	Tumor necrosis factor, alpha-induced protein 3	**2.5**	*0.0001*	1.6	*0.0127*
Vcam1	Vascular cell adhesion molecule 1	**5.0**	*0.0001*	1.1	*0.6424*
Serpinb2	Serine (or cysteine) peptidase inhibitor, B, 2	**-9.6**	*0.0001*	-1.6	*0.0109*

*****Only statistically significant (p<0.05) AAN-induced ≤-2 (in green) or ≥ 2 (in red) fold changes were considered.

******Compared to PBS control group.

Supplementary Table 3. Effect of Paquinimod on AAN-induced changes in inflammation and immunity blood gene expression in blood at Day 21

Gene symbol	Description	AAN *		AAN + Paquinimod	
		**Fold Change****	***p* Value****	**Fold Change****	***p* Value****
Apcs	Serum amyloid P-component	**3.0**	*0.0003*	-1.1	*0.0019*
Crp	C-reactive protein	**4.5**	*0.0004*	-3.1	*0.0746*
Csf2	Colony stimulating factor 2	**3.9**	*0.0072*	1.2	*0.9455*
Foxp3	Forkhead box P3	**2.4**	*0.0017*	2.4	*0.0002*
Ifna2	Interferon alpha 2	**2.9**	*0.0007*	1.0	*0.9711*
Ifnb1	Interferon beta 1	**2.5**	*0.0004*	1.4	*0.0456*
Il17a	Interleukin 17a	**3.5**	*0.0006*	-1.2	*0.4990*
Il2	Interleukin 2	**2.2**	*0.0009*	1.0	*0.9242*
Il23a	Interleukin 23a	**2.3**	*0.0106*	2.0	*0.0003*
Il5	Interleukin 5	**3.6**	*0.0001*	1.6	*0.1272*
Il6	Interleukin 6	**3.0**	*0.0048*	1.8	*0.0002*
Mbl2	Mannose-binding lectin (protein C) 2	**5.8**	*0.0006*	-2.1	*0.0514*
Mpo	Myeloperoxidase	**2.6**	*0.0061*	2.6	*0.0044*
Mx1	Myxovirus (influenza virus) resistance 1	**2.2**	*0.0001*	3.3	*0.0001*
Rag1	Recombination activating gene 1	**-3.2**	*0.0001*	-3.1	*0.0001*

*****Only statistically significant (p<0.05) AAN-induced ≤-2 (in green) or ≥ 2 (in red) fold changes were considered.

******Compared to PBS control group.

Supplementary Table 4. Effect of Paquinimod on AAN-induced changes in atherosclerosis-related aortic gene expression at Day 21.

		**AAN ***		**AAN + Paquinimod**	
**Gene symbol**	**Description**	**Fold Change****	***p* Value****	**Fold Change****	***p* Value****
Apoa1	Apolipoprotein A-I	**21.5**	*0.0001*	2.4	*0.0001*
Apob	Apolipoprotein B	**27.5**	*0.0001*	12.0	*0.0001*
Birc3	Baculoviral IAP repeat containing 3	**2.1**	*0.0006*	-1.1	*0.5215*
Ccl2	Chemokine (C-C motif) ligand 2	**2.8**	*0.0001*	1.2	*0.2066*
Ccl5	Chemokine (C-C motif) ligand 5	**2.6**	*0.0002*	1.0	*0.9266*
Ccr2	Chemokine (C-C motif) receptor 2	**2.1**	*0.0004*	-1.2	*0.0429*
Cd44	CD44 molecule (Indian blood group)	**2.6**	*0.0001*	1.3	*0.0009*
Cxcl1	Chemokine (C-X-C motif) ligand 1	**4.8**	*0.0046*	2.2	*0.0077*
Fga	Fibrinogen alpha chain	**25.6**	*0.0001*	10.0	*0.0001*
Fgb	Fibrinogen beta chain	**28.7**	*0.0001*	15.9	*0.0001*
Icam1	Intercellular adhesion molecule 1	**2.8**	*0.0001*	1.3	*0.0080*
Il1a	Interleukin 1, alpha	**5.4**	*0.0006*	1.7	*0.1557*
Il1b	Interleukin 1, beta	**5.8**	*0.0001*	-2.1	*0.0309*
Itgax	Integrin, alpha X	**3.0**	*0.0003*	1.5	*0.0042*
Msr1	Macrophage scavenger receptor 1	**2.1**	*0.0001*	-1.3	*0.0007*
Nyp	Neuropeptide Y	**5.7**	*0.0001*	9.0	*0.0112*
Ptgs1	Prostaglandin-endoperoxide synthase 1	**2.2**	*0.0001*	-1.2	*0.0231*
Sele	Selectin, endothelial cells	**2.1**	*0.0031*	-1.4	*0.1607*
Sell	Selectin, lymphocytes	**7.8**	*0.0001*	-1.4	*0.0584*
Selplg	Selectin, platelet (p-selectin) ligand	**2.4**	*0.0001*	-1.1	*0.5787*
Tnf	Tumor necrosis factor	**15.4**	*0.0030*	3.6	*0.1639*
Tnfaip3	Tumor necrosis factor, alpha-induced protein 3	**2.3**	*0.0001*	1.6	*0.0258*
Vcam1	Vascular cell adhesion molecule 1	**4.5**	*0.0001*	1.0	*0.8135*
Il2	Interleukin 2	**-2.9**	*0.0008*	-1.9	*0.0028*
Serpinb2	Serine (or cysteine) peptidase inhibitor, mbr 2	**-4.7**	*0.0002*	-1.2	*0.2618*

*Only statistically significant (p<0.05) AAN-induced ≤-2 (in green) or ≥ 2 (in red) fold changes were considered.

**Compared to PBS control group.

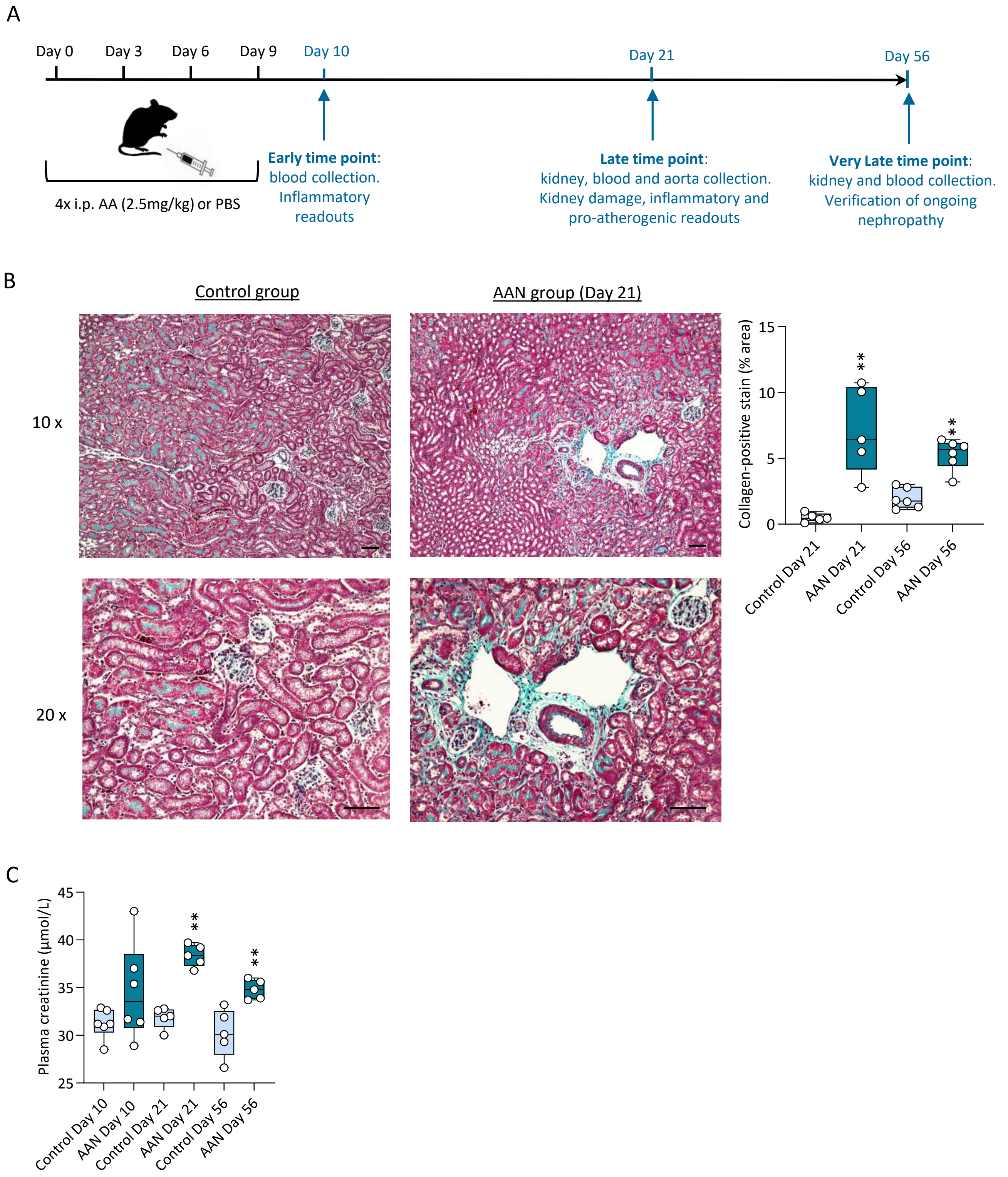

Supplementary Figure 1. Kidney damage and loss of function following repeated AA injections in mice
(A-C) C57BL/6J mice (at least n=5 per group) were injected intraperitoneally with AA (2.5mg/kg) or PBS on days 0, 3, 6, and 9 to induce chronic nephropathy. AAN was verified at Day 21 and Day 56 by the development of kidney fibrosis (B) and a significant elevation in plasma creatinine (C). B. Representative images of Masson trichrome stain of kidneys from a healthy mouse (left panels) and a mouse with chronic AAN (right) . Cytoplasm is stained red, nuclei are in dark brown, and collagen is stained blue, identifying renal fibrosis. Scale bars: 100 µm. Graph shows the percentage of collagen positive stain for each group (3 non-overlapping fields of view scored for each of 5 animals/group) **c**. Creatine measurements in plasma. Individual animal data points are shown and horizontal bars denote the median value for the group *, *p*<0.05, **, *p*<0.01, AAN vs Control, Mann-Whitney U test.

A

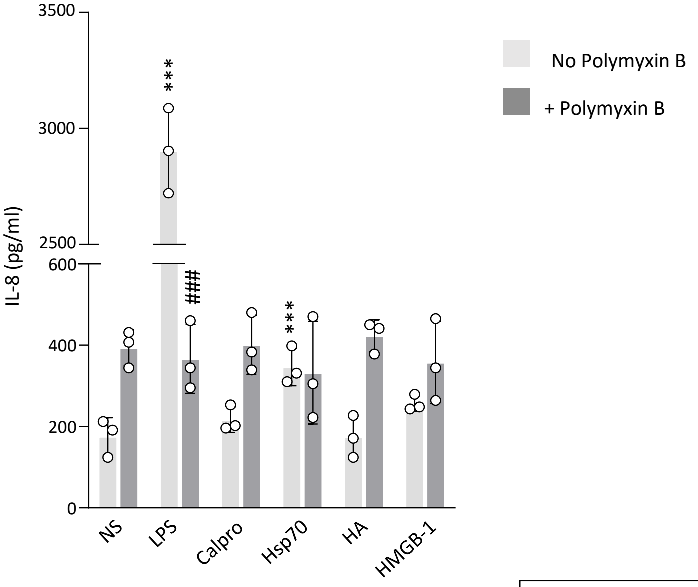

B

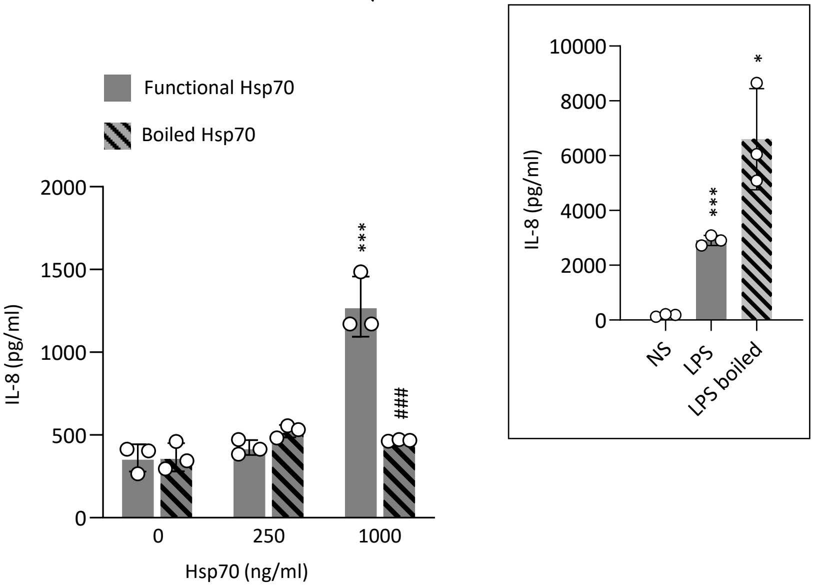

C

	Endotoxin concentration (EU)	Endotoxin concentration (pg/ml)
Hsp70	*non detectable*	*non detectable*
Calpro	*non detectable*	*non detectable*
HMGB-1	0.001	0.15
HA	*non detectable*	*non detectable*

Supplementary Figure 2. The preparations of CKD-associated DAMPs are not contaminated with significant amounts of endotoxin

A,B. Levels of IL-8 in culture supernatants from triplicate cultures of human Mono-Mac6 monocytes stimulated (18h, 37°C) with LPS (10 ng/ml) or the indicated DAMPs (1µg/ml, A or indicated concentrations, B) in the presence or absence of Polymyxin B (5µg/ml, A) or before and after boiling (95°C, 10 mins, B). *, *p*<0.05; **, *p*<0.01; ***, *p*<0.005. TLR ligand vs no stimulation (NS) or #, +Polymyxin B/Boiling vs No Polymyxin B/boiling, unpaired Student's t test. C. Endotoxin measurements in DAMP preparations by the LAL method.

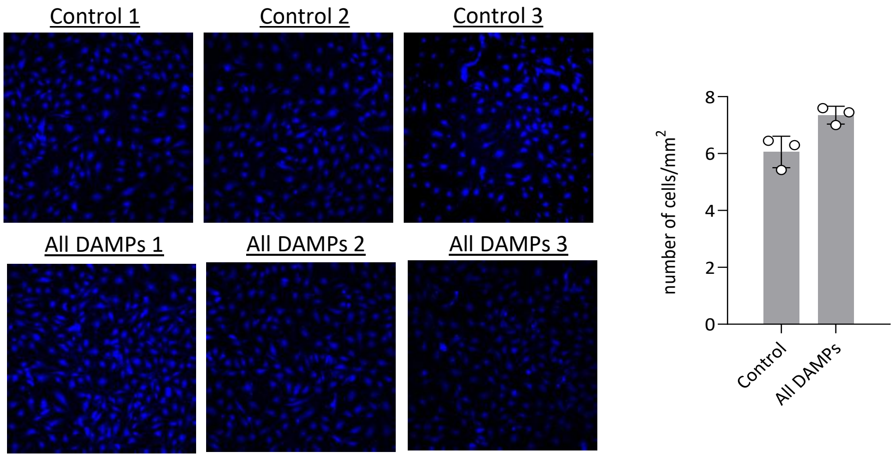

Supplementary Figure 3. Exposure to DAMPs does not reduce endothelial coverage or cell number
Triplicate cultures of human umbilical arterial endothelial cells were grown to confluence prior to stimulation or not (Control) for 5h with combined CKD-associated DAMPs (Calprotectin, Hsp70, HA, HMGB-1, 100 ng/ml each). Culture insets were stained with DAPI to highlight cell nuclei and cell numbers were counted for each triplicate using the ImageJ software. Plots show individual well measurements together with the mean (+/- SD) of cell density per group.

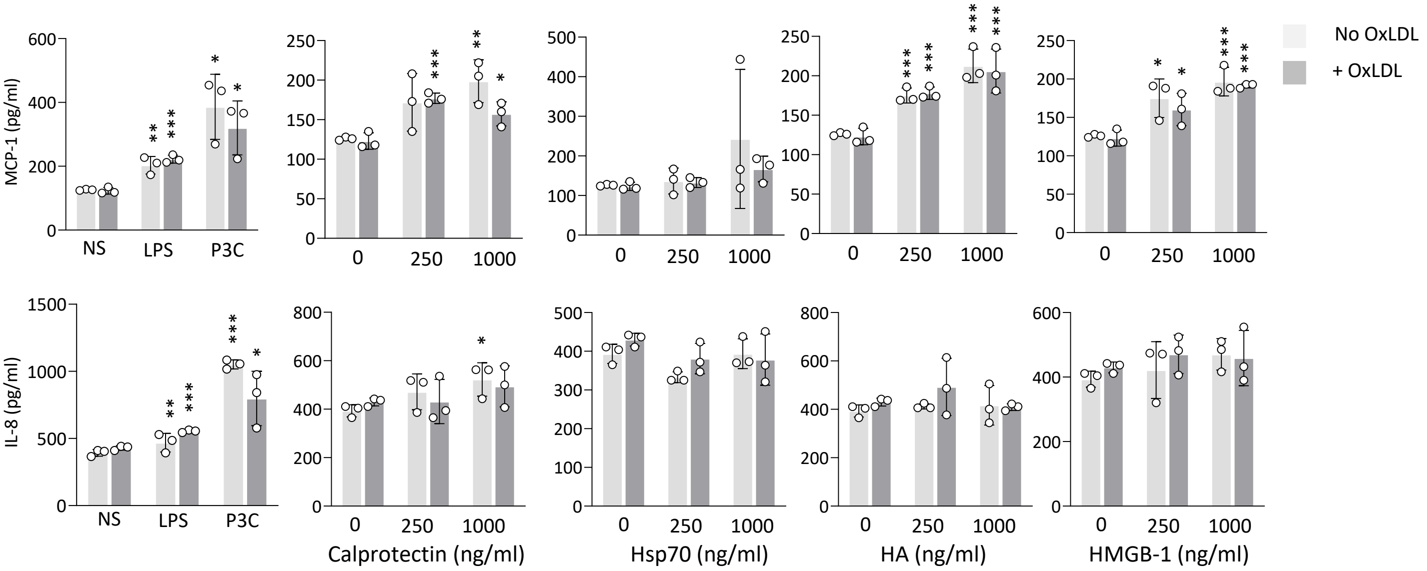

Supplementary Figure 4. Co-exposure to OxLDL does not affect CKD DAMP-induced pro-inflammatory mediator production by HAEC

Triplicate cultures of HAEC were stimulated (18h) with LPS (10 ng/ml), Pam_3CSK_4 (P3C, 500 ng/ml) or the indicated concentrations of DAMPs, in the presence or absence of OxLDL (10 µg/ml). Cytokine levels in culture supernatants are shown as mean +/- SD from one experiment representative of 3. *, *p*<0.05; **, *p*<0.01; ***, *p*<0.005, DAMP/LPS/P3C Stimulation vs no stimulation, unpaired Student's t test.

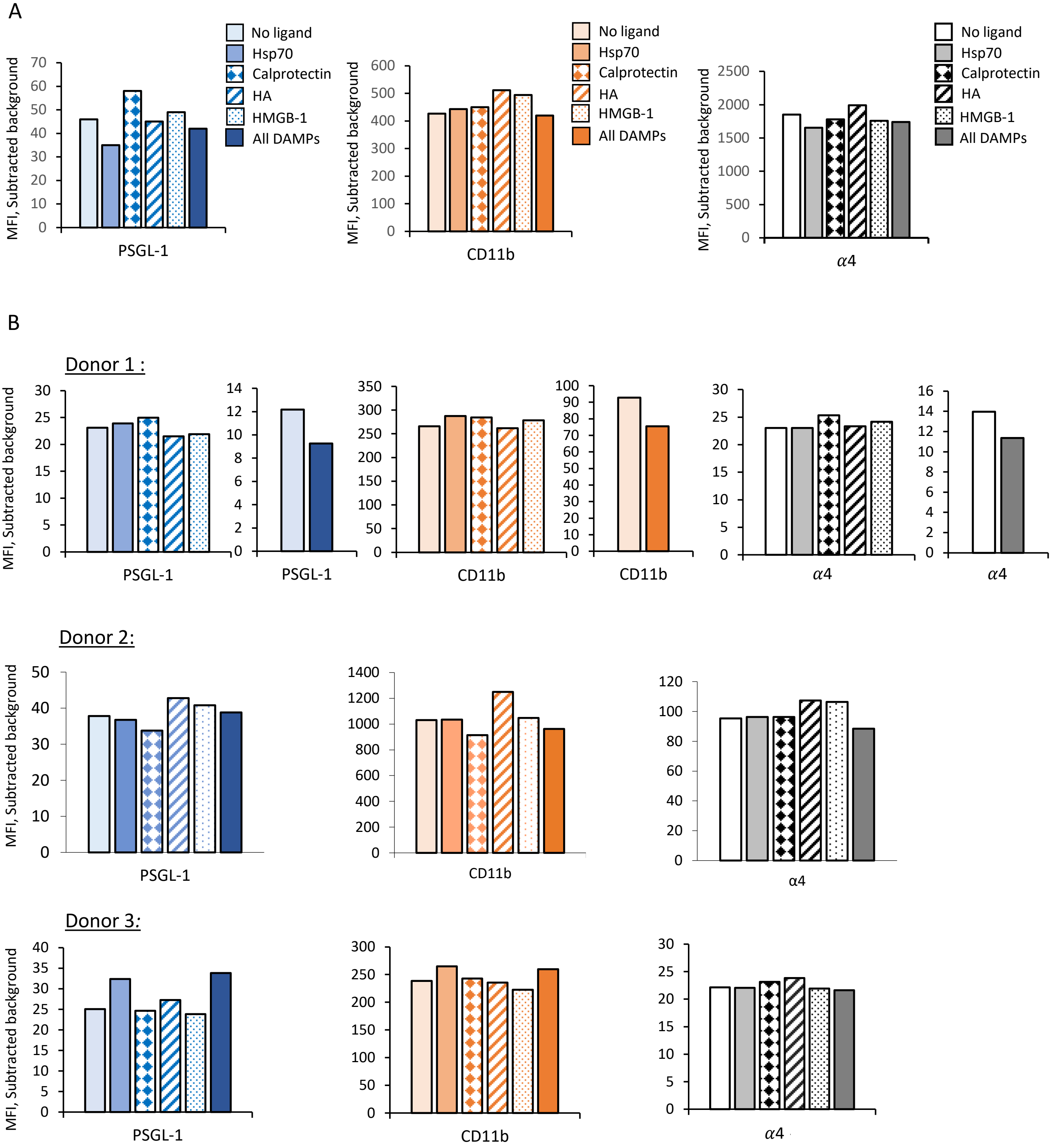

Supplementary Figure 5. The CKD-associated DAMPs do not significantly modulate expression of CD11b, integrin α4 and PSGL-1 by Mono-Mac6 or primary blood monocytes

Mono-Mac 6 cells (A) or PBMC (B) were stimulated for 48h with the indicated DAMPs (all at 1 μg/ml), alone or combined, prior to flow cytometry analysis for cell surface expression of CD11b, PSGL-1 and α4 integrin. In B, gated monocytes among PBMC were identified by their FSC/SSC scatter and positive expression of CD14. Results are mean fluorescence intensity (MFI) of at least 10,000 cells/conditions, with subtracted istotype control MFI.

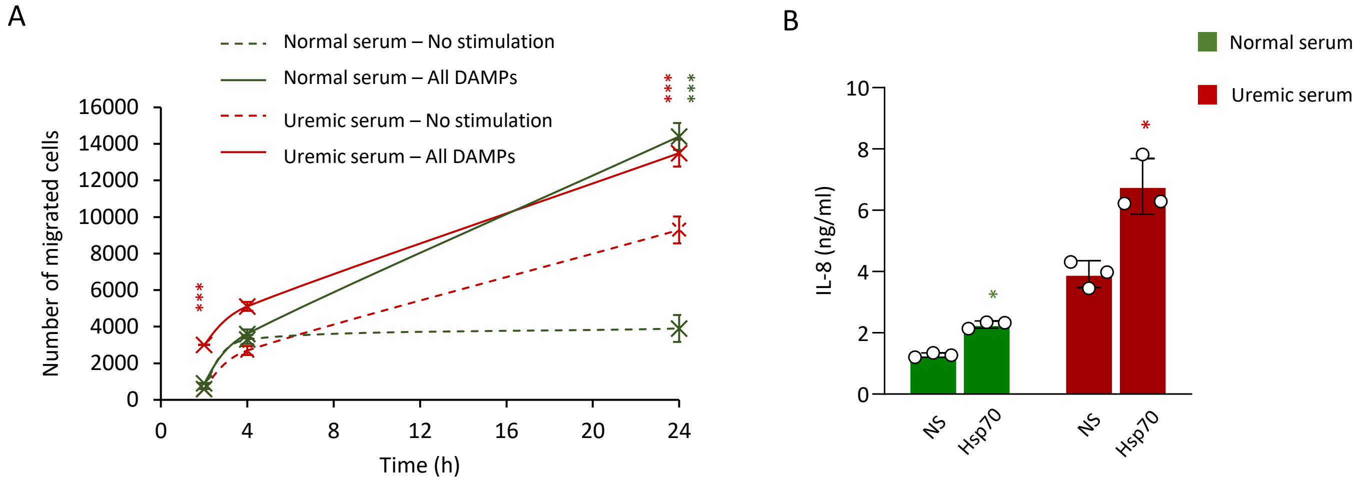

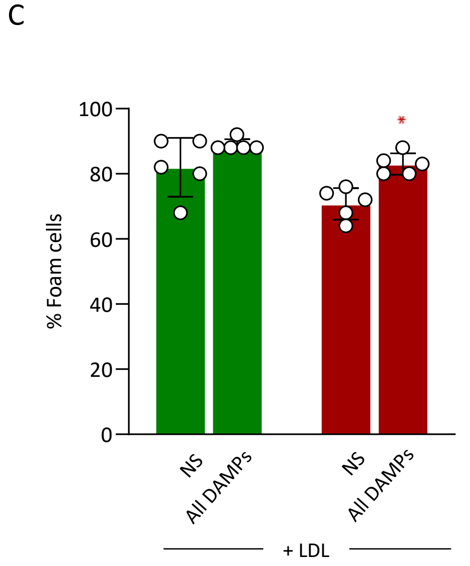

Supplementary Figure 6. Culture in uremic conditions does not negatively affect the effect of the DAMPs on monocyte migration and cytokine production or foam cell formation by macrophages

Triplicate cultures of Mono-Mac6 monocytes (A) or M-CSF-differentiated macrophages (B,C) were cultured for 48h in 25% normal AB serum (green) or serum from 5 pooled Stage 5 CKD patients (red) prior to assessing the effect of the indicated CKD-associated DAMPs on monocyte migration (A), IL-8 production by macrophages (B) and foam cell formation (c). These experiments were performed as previously described (Figures 4a, 5a and 6a). Open circles indicate triplicates (B) or independent fields of view (C) for each experimental condition. *, *p*<0.05; ***, *p*<0.005. DAMP stimulation vs no stimulation (NS), unpaired Student's t test.

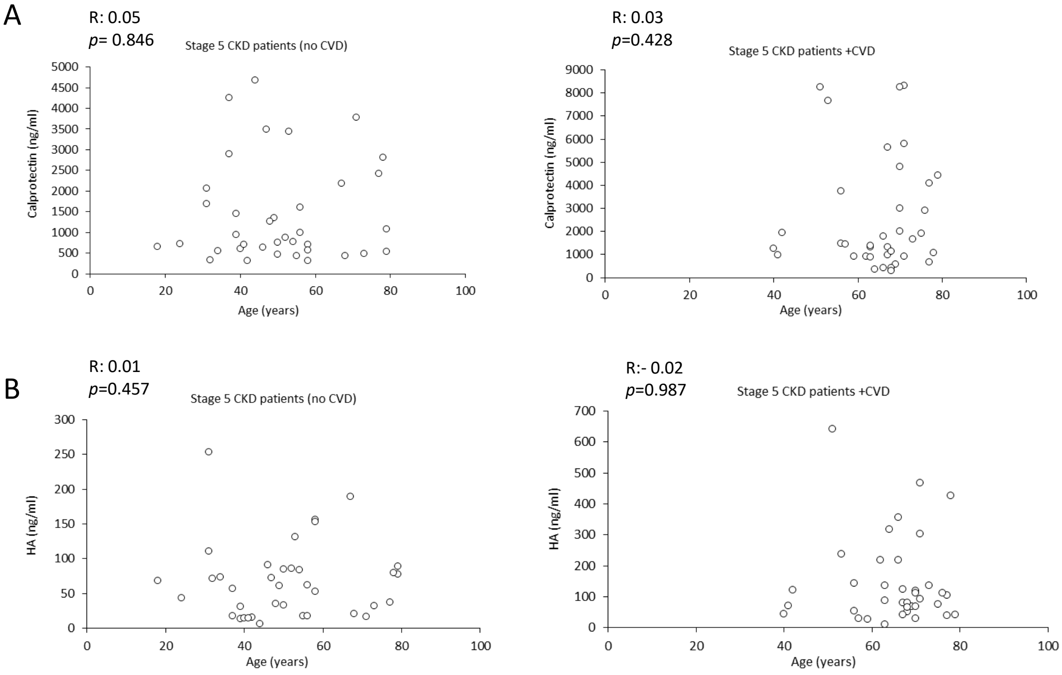

Supplementary Figure 7. Plasma levels of Calprotectin and HA in CKD patients with and without CVD are not associated with age

Concentrations of Calprotectin (A) or HA (B) in plasma from Stage 5 CKD patients with or without prior CVD diagnosis were determined by ELISA and monotonic association with patients age was determined by Spearman's rank correlation test (R, coefficient corelation). None of the associations were statistically significant.

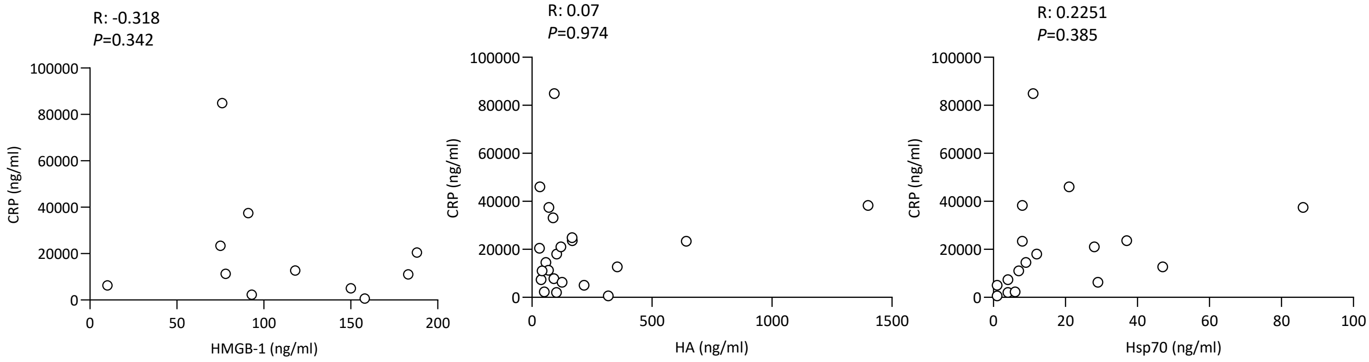

Supplementary Figure 8. Plasma levels of HMGB-1, HA and Hsp70 in CKD patients with CVD are not associated with CRP levels
Concentrations of HMGB-1, HA, Hsp70 and CRP in plasma from Stage 5 CKD patients with prior CVD diagnosis were determined by ELISA and monotonic association with between each DAMP and CRP was determined by Spearman's rank correlation test (R, coefficient corelation). None of the associations were statistically significant.

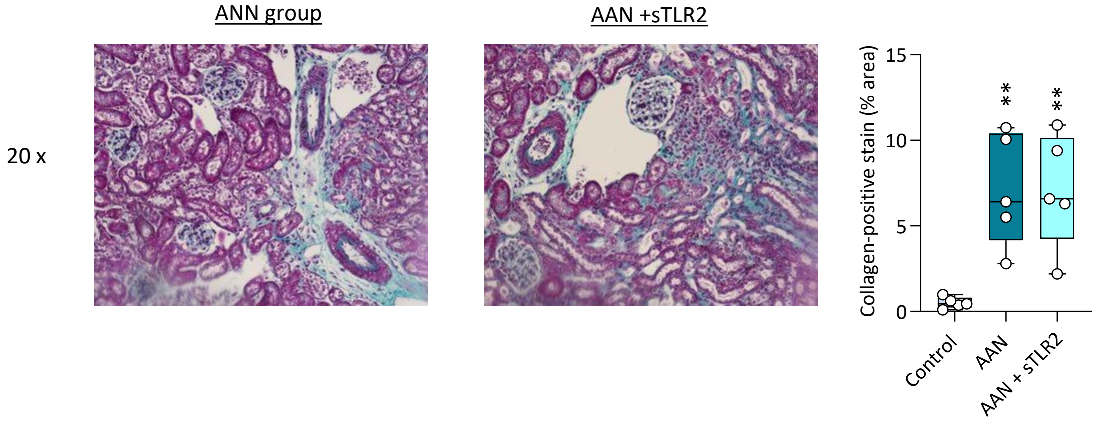

Supplementary Figure 9. Administration of sTLR2 does not reduce kidney damage in AAN mice
C57BL/6J mice (n=5 per group) were injected intraperitoneally with AA (2.5mg/kg) or PBS , in the presence or absence of sTLR2 (12.5µg/kg) on days 0, 3, 6, and 9 to induce chronic nephropathy. The extent of kidney fibrosis was evaluated at Day 21. Representative images of Masson trichrome stain of kidneys from a healthy mouse, a mouse with chronic AAN, and an AAN mouse with sTLR2 treatment. Cytoplasm is stained red, nuclei are in dark brown, and collagen is stained blue, identifying renal fibrosis. Scale bars: 100 µm. Graph shows the percentage of collagen positive stain for each group (3 non-overlapping fields of view scored for each of 5 animals/group). **, $p<0.01$, AAN or AAN + sTLR2 vs Control, Mann-Whitney U test.
